# New Insight into the Role of the Calvin Cycle: Reutilization of CO_2_ Emitted through Sugar Degradation

**DOI:** 10.1038/srep11617

**Published:** 2015-07-01

**Authors:** Rie Shimizu, Yudai Dempo, Yasumune Nakayama, Satoshi Nakamura, Takeshi Bamba, Eiichiro Fukusaki, Toshiaki Fukui

**Affiliations:** 1 Department of Bioengineering, Graduate School of Bioscience and Biotechnology, Tokyo Institute of Technology, 4259 Nagatsuta, Midori-ku, Yokohama 226-8501, Japan; 2 Department of Biotechnology, Graduate School of Engineering, Osaka University, 2-1 Yamadaoka, Suita, Osaka 565-0871, Japan

## Abstract

*Ralstonia eutropha* is a facultative chemolithoautotrophic bacterium that uses the Calvin–Benson–Bassham (CBB) cycle for CO_2_ fixation. This study showed that *R. eutropha* strain H16G incorporated ^13^CO_2_, emitted by the oxidative decarboxylation of [1-^13^C_1_]-glucose, into key metabolites of the CBB cycle and finally into poly(3-hydroxybutyrate) [P(3HB)] with up to 5.6% ^13^C abundance. The carbon yield of P(3HB) produced from glucose by the strain H16G was 1.2 times higher than that by the CBB cycle-inactivated mutants, in agreement with the possible fixation of CO_2_ estimated from the balance of energy and reducing equivalents through sugar degradation integrated with the CBB cycle. The results proved that the ‘gratuitously’ functional CBB cycle in *R. eutropha* under aerobic heterotrophic conditions participated in the reutilization of CO_2_ emitted during sugar degradation, leading to an advantage expressed as increased carbon yield of the storage compound. This is a new insight into the role of the CBB cycle, and may be applicable for more efficient utilization of biomass resources.

The Calvin–Benson–Bassham (CBB) cycle, employing ribulose-1,5-bisphosphate carboxylase/oxygenase (Rubisco) as a key CO_2_-fixing enzyme, is used for primary production by most plants, algae and various autotrophic microorganisms^1^. Because the CBB cycle is a highly energy-consuming pathway dependent on reductive assimilation of CO_2_, it is strictly repressed by regulation in several plants, algae and cyanobacteria when essential ATP and reducing equivalents are unavailable^2^,^3^,^4^,^5^. In facultative photoautotrophic purple bacteria, the CBB cycle operates not only during carbon assimilation under photoautotrophic conditions but also for dissipating excess reducing equivalents under photoheterotrophic conditions. Reg/Prr two-component signal transduction systems sense the redox states of cells and regulate global gene expression for various metabolisms including the CBB cycle in the purple non-sulphur bacteria *Rhodobacter sphaeroides* and *Rhodospirillum rubrum*^6^. Synthesis of Rubisco was completely repressed in *R. sphaeroides* under aerobic chemoheterotrophic conditions^6^. Algae and some chemolithoautotrophic bacteria grow mixotrophically by simultaneous function of autotrophic and heterotrophic metabolisms, which require light and adequate inorganic electron donors, respectively, along with organic compounds.

A Gram-negative facultative chemolithoautotrophic bacterium, *Ralstonia eutropha* (*Cupriavidus necator*) strain H16, can utilize various organic compounds such as sugars, organic acids, fatty acids and plant oils for heterotrophic growth. In autotrophic growth mode, the bacterium can utilize H_2_ as the energy source and fix CO_2_ by the CBB cycle^7^. Two sets of the enzymes in the CBB cycle are encoded in *cbb*_*c*_ and *cbb*_*p*_ operons in chromosome 2 and megaplasmid pHG1, respectively. The expression of the *cbb* genes is activated by a common transcriptional regulator, CbbR, encoded in the *cbb*_*c*_ operon, when the intracellular concentration of phosphoenolpyruvate (PEP) becomes low under autotrophic conditions^8^. Interestingly, it has been shown that partial derepression of the *cbb* genes occurs on some substrates, including fructose and citrate^9^, given that weak activities of Rubisco and other CBB-cycle enzymes were detected in the late heterotrophic growth phase^10^. However, to date, the effect of the partially derepressed CBB cycle on heterotrophic metabolism in *R. eutropha* has not been investigated.

*R. eutropha* H16 has been also known to accumulate poly(3-hydroxybutylate) [P(3HB)] as a storage compound under unbalanced growth conditions. It has been estimated that the P(3HB) accumulation has a role in survival under stress conditions. Bacterial P(3HB) and related polyhydroxyalkanoates (PHAs) have attracted industrial attention as possible alternatives to petroleum-based polymer materials because they are biodegradable thermoplastics produced from renewable carbon sources. A number of studies has focused on the biosynthesis of PHAs by *R. eutropha*, particularly in terms of the biosynthetic pathways and enzymes, and on metabolic engineering aimed at efficient production of PHA copolyesters from inexpensive biomass resources^11^,^12^,^13^,^14^,^15^. Recent transcriptome analyses of *R. eutropha* showed that the expression of the *cbb* genes was upregulated in the wild type strain H16 under nitrogen-deficient P(3HB) accumulation conditions^16^,^17^; however, it was downregulated in the PHA-negative mutant strain PHB^−^4 grown on gluconate^18^. Metabolome analysis of the H16 strain detected ribulose-1,5-bisphosphate (RuBP), a key metabolite specific to the CBB cycle, in cells of *R. eutropha* H16 cultured with fructose or octanoate^19^. Moreover, we detected slight incorporation of ^13^C atoms into P(3HB) during incubation of *R. eutropha* H16 in a fructose-containing medium supplemented with NaH^13^CO_3_, and confirmed that ^13^CO_2_ fixation was mediated by both the Rubiscos^17^. These observations strongly suggested some role of the CBB cycle in the heterotrophic P(3HB) biosynthesis from sugars.

We assumed that, when the CBB cycle is functional under heterotrophic conditions in the presence of sugars, it may act on fixation and reutilization of CO_2_ emitted by oxidative decarboxylation during the sugar degradation. Generally, microbial production of value-added compounds from sugars often accompanies marked loss of carbon because of decarboxylation. In particular, this phenomenon is critical for acetyl-CoA-derived compounds such as P(3HB) because one-third of the total carbon atoms in hexoses are lost as CO_2_ molecules emitted by the oxidative decarboxylation of pyruvate to acetyl-CoA. Considering the costs of harvest, transportation and saccharification of biomass-based polysaccharides, the loss of carbon during microbial sugar degradation cannot be negligible for the purpose of efficient utilization of biomass resources. Therefore, the design of metabolic pathways avoiding carbon loss is expected to be one way to establish more efficient bioprocesses. Chinen *et al*. have reported a pathway for efficient L-glutamate production from glucose by *Corynebacterium glutamicum* employing phosphoketolase (PKT) to bypass the CO_2_-releasing pyruvate dehydrogenase reaction^20^. However, to date, the reutilization of the decarboxylated carbon for the bioproduction of value-added compounds using the functions of the CBB cycle under heterotrophic conditions has not been studied.

Metabolomic approaches employing stable isotope labelling of metabolites are powerful tools for the analysis of metabolic dynamics^21^,^22^,^23^. Recently, Hasunuma *et al*. established the dynamic analysis of plant metabolism by isotope tracing of ^13^C from ^13^CO_2_ fed to *Nicotiana tabacum* leaves^24^. In this study, we constructed CBB cycle-inactivated mutants of *R. eutropha* and compared P(3HB) biosynthesis properties and ^13^C-labelling profiles with [1-^13^C_1_]-glucose, in which the ^13^C atom was emitted as ^13^CO_2_ through the Entner–Doudoroff (ED) pathway, with those of the parent strain. The result provided new insight into the function of the CBB cycle. The ‘gratuitously’ activated CBB cycle in *R. eutropha* under heterotrophic conditions played a role in fixation and reutilization of the carbon, generally wasted during sugar degradation, for biosynthesis of the storage polyester.

## Results

### Construction of CBB cycle-inactivated strains of *R. eutropha*

**Figure 1:**
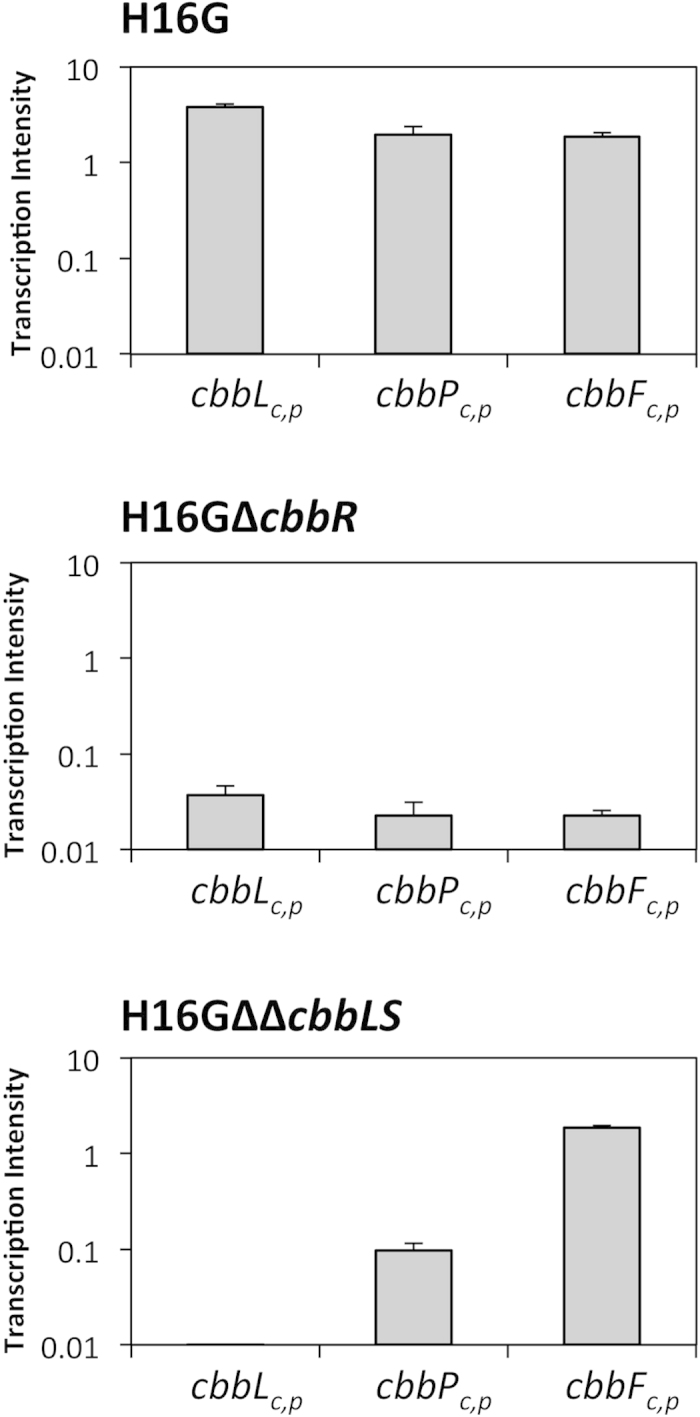
Relative gene expression levels of *cbbL*, *cbbP* and *cbbF* in *R. eutropha* strains H16G and the CBB cycle-inactivated strains (H16G∆*cbbR* and H16G∆∆*cbbLS*) grown on glucose.

Two CBB cycle-inactivated strains of *R. eutropha* were constructed using the glucose-assimilating recombinant strain H16G (renamed from the previously constructed strain H16∆*nagR*_*nagE*-G793C^25^) as a host strain. In the mutant strain H16G∆∆*cbbLS*, both *cbbLS*_*c*_- and *cbbLSp*-encoding Rubisco enzymes were deleted from chromosome 2 and pHG1, respectively, by homologous recombination. Note that this strain retains other *cbb* genes involved in regeneration of RuBP in the CBB cycle. Another mutant strain, H16G∆*cbbR*, was constructed by deletion of *cbbR* on chromosome 2, encoding a common transcriptional activator for the two *cbb* operons. It has been reported that a *cbbR*-deleted strain of *R. eutropha* was incapable of growing autotrophically owing to insufficient induction of the *cbb* genes^26^. Indeed, qRT-PCR analysis demonstrated that expression levels of *cbbL* (encoding Rubisco large subunit), *cbbP* (encoding phosphoribulokinase) and *cbbF* (encoding fructose-1,6-bisphosphatase I/sedoheptulose-1,7-bisphosphatase) in H16G∆*cbbR* incubated with glucose were approximately one-hundredth compared to those in the parent strain H16G (Fig. 1). H16G∆∆*cbbLS* showed higher expression of *cbbP* and *cbbF* than H16G∆*cbbR*, as expected.

### Metabolomics of the *R. eutropha* strains producing P(3HB) from [1-^13^C_1_]-glucose

**Figure 2:**
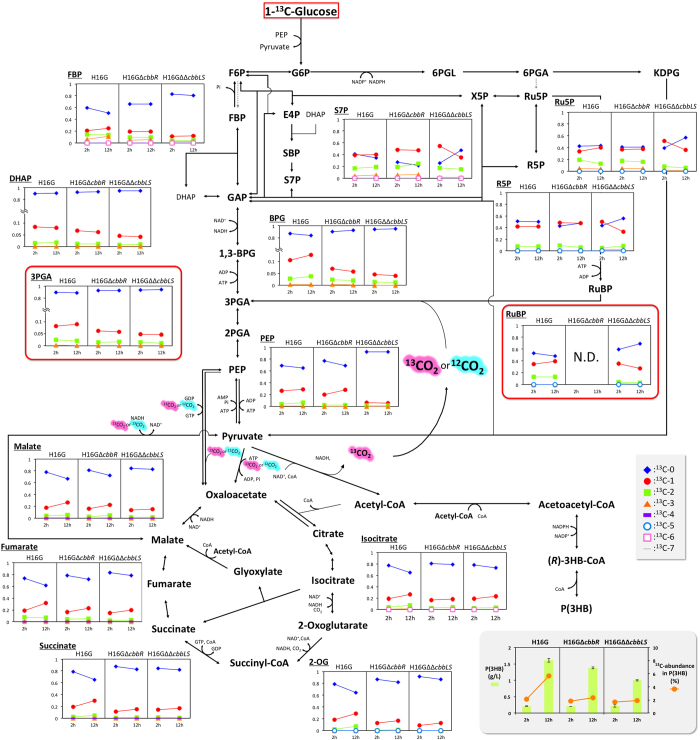
Time-dependent changes of mass distribution of metabolites in central metabolisms, as well as production of and ^13^C abundance in P(3HB) with *R. eutropha* H16G and H16G∆*cbbR*, and H16G∆∆*cbbLS* incubated with [1-^13^C_1_]-glucose. Abbreviations are shown in supplementary Table S1.

The *R. eutropha* strains were first cultivated in a nutrient-rich medium and the grown cells were then incubated in a nitrogen-free mineral salt medium containing [1-^13^C_1_]-glucose. The cellular metabolites were extracted from the cells incubated with [1-^13^C_1_]-glucose for 2 h and 12 h and subjected to metabolomic analysis to detect the incorporation of ^13^C into each of the metabolites. Analysis using reversed-phase ion-pair liquid chromatography coupled with triple-quadrupole mass spectrometry (RP-IP-LC/QqQ-MS) was able to determine the ^13^C-labelling ratios for 31 metabolites including sugar phosphates, organic acids, amino acids and CoA thioesters. Unexpectedly, high incorporation of ^13^C in free coenzyme A (CoA) was observed during the incubation with [1-^13^C_1_]-glucose for all the strains examined, and the profile was nearly the same as those of the detectable CoA-thioesters (acetyl-CoA, butyryl-CoA, succinly-CoA, 3-hydroxybutyryl-CoA and crotonyl-CoA). The ^13^C abundances in acyl moieties of the CoA thioesters could not be precisely determined, owing to ^13^C accumulation in the CoA backbone, so that the results for CoA-thioesters were not further used. The mass distributions of 26 metabolites are shown in supplementary Table S2, and those of 14 metabolites in sugar metabolism are shown along with the metabolic pathways in Fig. 2. The changes in abundance of [^13^C_1_]-derivatives were useful for evaluating the level of ^13^C incorporation, given that derivatives multiply labelled with 2 or more ^13^C atoms were generally present at low abundance (<7%) except for a few metabolites.

The ^13^C atom was slightly but significantly incorporated into 3-phosphoglycerate (3PGA) (*p* < 0.01), a product of Rubisco-mediated CO_2_ fixation, in H16G incubated with [1-^13^C_1_]-glucose. In contrast, the abundance of the ^13^C-containing isotopomers of 3PGA was slightly decreased in H16G∆*cbbR* and H16G∆∆*cbbLS*. Similar tendencies were observed for dihydroxyacetone phosphate (DHAP), 1,3-bisphosphoglycerate (1,3-BPG) and fructose-1,6-bisphoaphate (FBP), although [^13^C_1_]-phosphoenolpyruvate (PEP) in H16G∆*cbbR* increased. The abundances of the [^13^C_1_]-isotopomer of 3PGA, 1,3-BPG and DHAP in H16G were approximately 10%, which was apparently lower than those of PEP and FBP (~20%). This observation suggested slow turnover of the triose phosphates in *R. eutropha* under the conditions studied.

Significant and interesting differences among the three strains were observed for RuBP, a metabolite specific to the CBB cycle as a substrate for Rubisco. RuBP was detected not in H16G∆*cbbR* but in H16G and H16G∆∆*cbbLS*. The abundance of [^13^C_1_]-RuBP in H16G harbouring intact *cbb* operons increased during incubation with [1-^13^C_1_]-glucose from 2 to 12 h, whereas the abundance decreased in H16G∆∆*cbbLS* lacking Rubiscos. Ribulose-5-phosphate (Ru5P), ribose-5-phosphate (R5P) and sedoheptulose-7-phosphate (S7P) were detected in all strains. The [^13^C_1_]-isotopomers of these pentose phosphates and heptose phosphate had already constituted 40–60% of the total metabolites after incubation with [1-^13^C_1_]-glucose for 2 h. The abundances of the [^13^C_1_]-isotopomers then decreased in H16G∆∆*cbbLS* but increased in H16G during further incubation, as seen for RuBP in the respective strains. ^13^C accumulation into these sugar phosphates was relatively stable in H16G∆*cbbR* after rapid increase until 2 h.

Several carboxylic acids in the tricarboxylic acid (TCA) cycle showed similar changes in ^13^C accumulation among the three strains. The abundances of the [^13^C_1_]-isotopomers slightly increased during incubation with [1-^13^C_1_]-glucose from 2 to 12 h, with the rates of increase in H16G higher than those in the other two mutants. The analysis also detected several amino acids in the extracts, as shown in supplementary Table S2, and the changes in ^13^C accumulation were similar to those of the related carboxylic acids in the respective strains. For example, the profiles observed for serine and glutamate were very similar to those for 3PGA and 2-oxoglutarate, respectively. The abundances of the ^13^C-labelled isotopomers of the most detectable amino acids were higher in H16G than in H16G∆*cbbR* and H16G∆∆*cbbLS*.

### ^13^C incorporation into P(3HB) synthesized from [1-^13^C_1_]-glucose

The abundance of the [1-^13^C_1_] glucose-derived ^13^C atom in P(3HB) was determined by gas chromatography-mass spectrometry (GC-MS) analysis of methyl 3-hydroxybutyrate formed by methanolysis of the polymer. The ^13^C abundances in P(3HB) synthesized by H16G∆*cbbR* and H16G∆∆*cbbLS* (2.3% and 1.9%, respectively) were slightly higher than the natural abundance (1.1%), whereas that in P(3HB) synthesized by H16G was intriguingly higher (5.6%) (Fig. 2 and Table 1). These observations indicated that the CBB cycle in H16G was responsible for the incorporation of ^13^C derived from [1-^13^C_1_] glucose into P(3HB).

### Carbon yields P(3HB) on glucose by *R. eutropha* strains

The carbon yields of the conversion of P(3HB) to glucose were determined for the three *R. eutropha* strains by two-step cultivation. Because small amounts of P(3HB) (approximately 7–10 wt%) had been accumulated within the cells after the first step of cultivation in the rich medium, the yield during the second step of cultivation was calculated from the linear relationship between P(3HB) production and glucose consumption observed during several independent cultivations for various incubation periods. As shown in Table 1, the P(3HB) yields of H16G∆*cbbR* and H16G∆∆*cbbLS* were approximately half of the theoretical yield of 0.53 g-P(3HB)/g-glucose, and that of H16G was determined to be 1.2-fold higher than those of the two mutant strains lacking *cbbR* or *cbbLSs*.

## Discussion

The ^13^C abundance in P(3HB) synthesized by H16G∆∆*cbbLS*, in which the CBB cycle cannot function owing to the lack of Rubiscos, was slightly increased from the natural abundance of 1.1% to 1.9% after incubation with [1-^13^C_1_]-glucose for 12 h. Considering that *R. eutropha* H16 does not possess phosphofructokinase and 6-phosphogluconate dehydrogenase in the Embden–Meyerhof (EM) and pentose phosphate (PP) pathways, respectively, the ED pathway is a unique sugar degradation pathway, so that [1-^13^C_1_]-pyruvate and unlabeled glyceraldehyde 3-phosphate (GAP) were produced from [1-^13^C_1_]-glucose. When [1-^13^C_1_]-pyruvate was converted to acetyl-CoA by decarboxylation or anaplerosis and the successive TCA cycle, ^13^CO_2_ was emitted and unlabeled acetyl-CoA was produced, even in the case of fixation of H^13^CO_3_^−^ by anaplerotic reactions. Thus, the ^13^C atom derived from [1-^13^C_1_]-glucose was not incorporated into P(3HB) through the first round of the ED pathway (supplementary Fig. S1). Besides decarboxylation, [1-^13^C_1_]-pyruvate could be converted via the EM pathway, resulting in distribution of the ^13^C atom at the 1-position of triose phosphates, followed by formation of [3-^13^C_1_]-, [4-^13^C_1_]- or [3,4-^13^C_2_]-fructose-6-phosphate (F6P). While, [1-^13^C_1_]-F6P was formed by 6-phosphorylation and isomerization of [1-^13^C_1_]-glucose. These ^13^C atoms were distributed at the 1-, 2-, 3- and/or 4-positions of F6P through interconversion of C_4_-C_7_ sugar phosphates by a non-oxidative branch of the PP pathway [supplementary Fig. S5(A) and (C)] or RuBP regeneration steps in CBB cycle [supplementary Fig. S5(B) and (D)]. The ^13^C-labelled F6P molecules were converted to acetyl-CoA containing ^13^C at the 1 and/or 2 positions by the ED pathway and oxidative decarboxylation of pyruvate. Thus, the ^13^C atoms could be incorporated into P(3HB) without Rubisco-mediated CO_2_ fixation (supplementary Fig. S2). This event explained the slight increase of ^13^C abundance in P(3HB) to 1.9% in the H16G∆∆*cbbLS* strain.

When the CBB cycle in the H16G strain operated during P(3HB) biosynthesis from glucose, the CO_2_ molecules emitted by oxidative decarboxylation of pyruvate had a chance to couple with RuBP by the Rubisco-mediated reaction. As shown in supplementary Fig. S3, the complete CBB cycle likely established a new pathway for conversion of the ^13^C-labelled RuBP derived from [1-^13^C_1_]-glucose to ^13^C-labelled acetyl-CoA, even when ^12^CO_2_ was fixed. It was expected that the fixation of ^13^CO_2_, generated from [1-^13^C_1_]-pyruvate, by Rubisco enriched ^13^C atoms in the sugar phosphates via interconversion of sugar phosphates, leading to incorporation of more ^13^C into P(3HB) via ^13^C-enriched acetyl-CoA (supplementary Fig. S4). Indeed, the ^13^C abundance in P(3HB) synthesized from [1-^13^C_1_]-glucose by H16G increased to 5.6%. This observation demonstrated the actual fixation of CO_2_ emitted by decarboxylation during glucose degradation by the CBB cycle, and incorporation of the fixed carbon into P(3HB) in *R. eutropha*.

Metabolomic analysis revealed the increase of the [^13^C_1_]-isotopomers of most sugar phosphates, including RuBP and 3PGA, in the H16G strain during incubation with [1-^13^C_1_]-glucose (Fig. 2). This observation provided strong evidence for the actual flux of the CBB cycle with fixation of the [1-^13^C_1_]-glucose-derived ^13^CO_2_. In contrast, the [^13^C_1_]-isotopomers decreased in H16G∆∆*cbbLS*. The presence of intracellular RuBP without increase of ^13^C was reasonable, because RuBP could be generated by phosphoribulokinase (CbbP), but not be converted owing to the lack of Rubiscos in this strain. The absence of RuBP in H16G∆*cbbR* was consistent with the too low expression of *cbbP* and other *cbb* genes caused by the deletion of the transcriptional activator CbbR. However, the abundances of the [^13^C_1_]-isotopomers for many metabolites in H16G∆*cbbR* tended to show changes between those in H16G and H16G∆∆*cbbLS*. These results suggested that the subtly expressed *cbb* genes even in the absence of the activator CbbR, as shown by qRT-PCR, which resulted in the slightly higher ^13^C-abundance in P(3HB) synthesized from [1-^13^C_1_]-glucose by H16G∆*cbbR* (2.3%) than by H16G∆∆*cbbLS* (1.9%).

**Figure 3:**
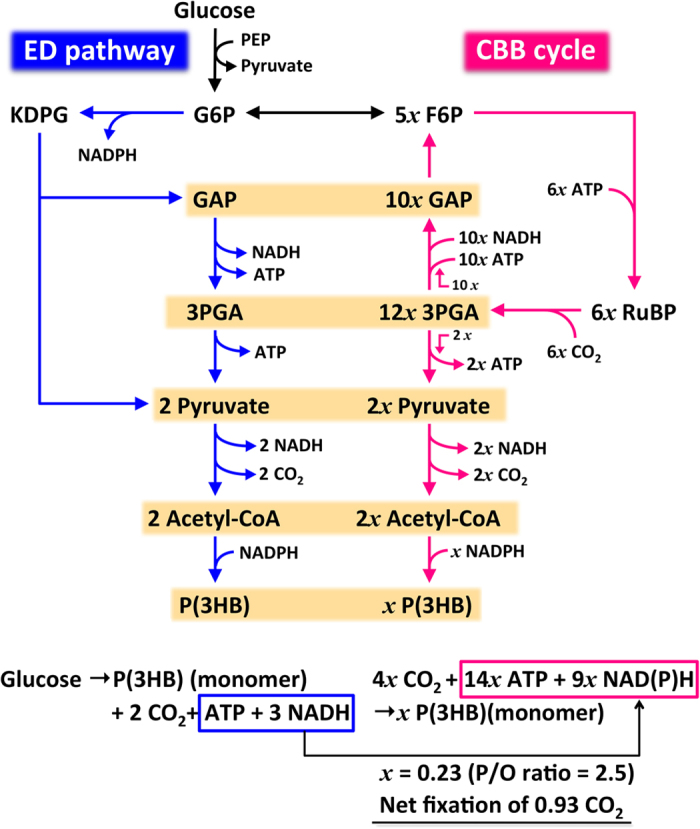
P(3HB) biosynthesis from glucose in *R. eutropha* H16G through an integrated pathway with the ED pathway and CBB cycle.

By *R. eutropha* H16G, one molecule of glucose is converted to P(3HB) monomer [(*R*)-3HB-CoA] along with two molecules of CO_2_ and surplus energy and reducing equivalents via the ED pathway, as shown as the left-hand equation in Fig. 2. The conversion of CO_2_ to P(3HB) monomer by the combination of the CBB cycle and P(3HB) biosynthesis is shown as the right-hand equation in Fig. 2. On the assumption that NADH is equivalent to NADPH and alternatively acted as an electron donor for generation of 2.5 ATP through aerobic respiration (P/O ratio = 2.5^27^), it was estimated that the energy and reducing equivalents released during conventional P(3HB) synthesis from glucose correspond to those essential for fixation of 0.93 CO_2_ into P(3HB) (Fig. 3). Thus, when the CBB cycle is functional during heterotrophic P(3HB) biosynthesis not associated with cell growth, 0.23 molecules of 3HB monomer are expected to be additionally obtained from the fixed 0.93 molecules of CO_2_ (46.5% recovery from 2 molecules of CO_2_ emitted from 1 molecule of glucose), leading to increase of the P(3HB) yield to 123%. This calculation agreed well with the finding that *R. eutropha* H16G produced P(3HB) in 117–122% yield relative to H16G∆*cbbR* and H16G∆∆*cbbLS* (Table 1). Apparently, the active CBB cycle under heterotrophic conditions was an advantage in P(3HB) production for *R. eutropha*. However, the net increase of ^13^C abundance in P(3HB) synthesized from [1-^13^C_1_]-glucose by H16G was 4.5% of the natural abundance (1.1%), which was lower than the 7.8% simply estimated from the fixation of 0.93 CO_2_ in the two CO_2_ molecules derived from [1-^13^C_1_]-glucose. We initially supposed that this discrepancy was due to stable carbon isotope discrimination by Rubisco, but this reason is unlikely because the ratio of reaction rate toward ^12^CO_2_ to that toward ^13^CO_2_ (*ε*-values) for Rubisco from *R. eutropha* was only 1.9% by *in vitro* assay^28^. Considering that the yields of P(3HB) by the *R. eutropha* strains were lower than the theoretical maximum yield, some acetyl-CoA molecules were inferred to have been completely degraded to CO_2_ by the TCA cycle to obtain energy under aerobic conditions for maintaining various cellular functions. This process would reduce the abundance of ^13^CO_2_ within cells, reflecting the lower-than-expected ^13^C abundance in P(3HB). The turnover of the TCA cycle in the P(3HB) accumulation phase not associated with cell growth was supported by the increase of the ^13^C-labelled isotopomers of intermediate acids in the TCA cycle in H16G (Fig. 2).

Recently, Guadalupe-Medina *et al*. have reported that functional expression of type II Rubisco and phosphoribulokinase in *Saccharomyces cerevisiae* established a bypass for glucose degradation not producing excess NADH and resulting in reduced glycerol formation during bioethanol production under anaerobic conditions^29^. In the present study, we found that the CBB cycle in *R. eutropha* plays a role in fixation of CO_2_ emitted by oxidative decarboxylation during sugar degradation and reutilized the fixed CO_2_ as a source of P(3HB). It should be further noted that this novel function of CBB cycle was expressed under aerobic heterotrophic conditions, differently from those of the CBB cycle in purple non-sulphur bacteria under anaerobic photoheterotrophic conditions and chemoautotrophic bacteria under mixotrophic conditions. There are expected to be three important factors for this: heterotrophic derepression of the CBB cycle by an unique intercellular PEP sensor CbbR^8^ at a probable low intracellular concentration of PEP attributed to P(3HB) formation, a high ratio of carboxylase activity to oxygenase activity (τ value) of 75 for the red-type Rubisco from *R. eutropha*^30^ and simple modulation of Rubisco activity by a AAA^+^ protein CbbX specific for red-type Rubiscos^31^. The more accumulation of the storage compound may be beneficial for survival in natural habitats. Moreover, the present results suggested that the CBB cycle, under heterotrophic conditions, could raise base yields of useful compounds by reutilization of the carbon atom emitted from carbon sources, when the reducing equivalents obtained by heterotrophic metabolisms were greater than those required for biosynthesis of the end products. Although the generation and consumption of reducing equivalents were equally balanced in typical anaerobic fermentation such as production of ethanol and 1-butanol, surplus reducing equivalents are available in biosynthesis of some bioproducts; for example, P(3HB), optically active 3-hydroxybutyrate and 2-propanol. The functional integration of carbon-fixing enzyme/pathways into the metabolic networks of industrial microorganisms may be a useful strategy for avoiding the loss of biomass-derived carbons in such cases.

## Materials and Methods

### Construction of disruption plasmids and recombinant strains

General cultivation of bacterial strains and construction plasmids pK18ms∆*cbbLSc* and pK18ms∆*cbbLSp* for deletion of *cbbLS*_*c*_ and *cbbLS*_*p*_, respectively, have been reported in previously^17^. A plasmid pK18ms∆*cbbR* for deletion of *cbbR* from chromosome 2 of *R. eutropha* H16 was constructed as follows. First, upstream and downstream regions (1 kbp) of *cbbR* were individually amplified by PCR with genomic DNA of *R. eutropha* H16 as a template and primer sets of cbbR-up5’ (GCTCTAGAAGCCATTTGGCAATCACGCGGA)/cbbR-up3’ (TCCTGGAACCGGGCGGTTGGGGGCGGCTTTGGAT) and cbbR-down5’ (CCAACCGCCCGGTTCCAGGAGGGTTGGCTGGGATT)/cbbR-down3’ (GGAATTCTGGTAGCGGCGTTGTCATACACAT), respectively. The underlined sequence showed the restriction sites for XbaI and EcoRI, respectively. The second PCR with the amplified fragments using cbbR-up5’/cbbR-down3’ primers gave a fused fragments of upstream and downstream regions of *cbbR*. The resulting fragment was digested by EcoRI and XbaI and then ligated with pK18mobsacB^32^ at the corresponding sites to obtain pK18ms∆*cbbR*.

Transconjugation of the mobilizable plasmids from *E. coli* S17-1 to *R. eutropha* H16G and isolation of strains generated by pop in-pop out recombination using pK18mobsacB-based suicide plasmids were preformed as described previously^12^,^14^. The strains H16G∆∆*cbbLS* and H16G∆*cbbR* were obtained by double deletion of *cbbLS*_*c*_ and *cbbLS*_*p*_, and single deletion of *cbbR* in *R. eutropha* H16G, respectively.

### P(3HB) production by two-step cultivation and determination of the carbon yield

*R. eutropha* H16G, H16G∆*cbbR*, and H16G∆∆*cbbLS* strains were firstly cultivated in 100 ml of a nutrient rich medium in 500 ml flask, with reciprocal shaking (117 strokes/min) for 14 ~ 15 h at 30 °C. The grown cells in 40 ml of the culture broth were harvested by centrifugation (5,000 *g*, 4 °C for 3 min), and washed with a mineral salt solution (9 g/l Na_2_HPO_4_ • 12 H_2_O, 1.5 g/l KH_2_PO_4_ in deionized water). The cell pellet was resuspended with 40 ml of a nitrogen-free MB medium composed by 9 g/l Na_2_HPO_4_ • 12 H_2_O, 1.5 g/l KH_2_PO_4_, 0.2 g/l MgSO_4_ and 40 μl of trace element solution^33^ and 5 g/l naturally-labeled glucose in a 200 ml flask. The cell suspension was further incubated with reciprocal shaking (128 strokes/min) at 30 °C as the second step cultivation for P(3HB) biosynthesis.

After the incubation for various times (2 h ~ 30 h), the cells and supernatant were separated by centrifugation (5,000 *g*, 10 min, 4 °C). Glucose concentration in the supernatant was measured by glucose oxidase method using a glucose kit GLU-NEO (SHINO-TEST, Tokyo, Japan). The recovered cells were washed with cold deionized water, and then lyophilized. The cellular P(3HB) content was determined by gas chromatography after methanolysis of the dried cells in the presence of 15% (v/v) sulfuric acid in methanol as described previously^33^. The carbon yield of P(3HB) was calculated from the amount of the produced P(3HB) and consumption of glucose during the cultivation.

### Quantitative real-time PCR (qRT-PCR)

The two-step cultivation of the *R. eutropha* strains was performed as mentioned above, and total RNA was isolated from the cells incubated with glucose under a nitrogen-depleted condition (the second stage) for 2 h by using RNeasy Midi Kit (Qiagen, Valencia, CA, USA). cDNA was synthesized by using ReverTra Ace qPCR RT Master Mix with gDNA Remover (TOYOBO, Osaka, Japan) according to the manufacturer’s instruction. Real-time PCR was performed by using Thunderbird SYBR qPCR Mix (TOYOBO, Osaka, Japan) with Thermal Cycler Dice Real Time System (Takara Bio, Otsu, Japan). The reaction conditions were: 1 min at 95 ^o^C, 40 cycles of 10 s at 95 ^o^C and 30 s at 60 ^o^C). *bfr2* (*h16_A0328*) was used as an inner control gene^17^, and primer sequences are listed in supplementary Table S3. The primers for amplification of a region in *cbbL*, *cbbP* and *cbbF* were designed to bind to both the copies in *cbb*_*c*_ and *cbb*_*p*_ operons.

### ^13^C-labeling of metabolites and P(3HB) using [1-^13^C_1_]-glucose

*R. eutropha* strains were cultivated by the two-step cultivation, where the second step was done with 5 g/l [1-^13^C_1_]-glucose (1-^13^C 98-99%, Cambridge Isotope Laboratories, Andover, MA, USA). 5-ml portion of the culture broth were taken at 2 h or 12 h for metabolomics analysis as follows. The remaining culture broth was used to determine the cellular content of P(3HB) as described above, and the ^13^C-abundance in the resulting P(3HB) was measured by GC-MS according to the procedure described previously^17^.

### Metabolite extraction and sample preparation

The 5-ml portion of the culture broth was put into 30 ml of 60% (v/v) methanol pre-cooled in ethanol/dry ice bath in a 50-ml centrifuge tube. The quenched cells were immediately recovered by centrifugation (8,000 *g*, 5 min, –8 °C), and dried *in vacuo*. The extraction of the intracellular metabolites from the dried cells was performed according to the procedure previously reported^34^ with slight modifications. Three milliliters of methanol/water/chloroform (2.5:1:1) was added into the dried cells in the 50-ml tube, and vigorously shaken by using vortex mixer for 30 s. The cell suspension was kept in –80 °C for 30 min, and then allowed to thaw at –30 °C and sonicate for 1 min. This cycle of freezing, thawing, and sonicating of the cells at the low temperatures was repeated three times for efficient extraction of the intercellular metabolites without decomposition. The precipitated proteins were removed by centrifugation at 16,000 *g*, 4 °C for 30 min. The two milliliters of the resulting supernatant was divided to 1 ml each in two micro tubes, and mixed with 200 μl of ultrapure water. The mixture was centrifuged, and the 1 ml aliquots of the polar phase were combined and concentrated using a concentrator VC-36S (Taitech Co., Tokyo, Japan) to a final volume of approximately 50 μl. The 20 μl portions of the extracts were taken and stored at –80 °C as metabolite extracts to be analyzed.

### Mass spectrometry

The metabolite extracts were analyzed byRP-IP-LC/QqQ-MS by using a Nexera UHPLC system equipped with LCMS 8030 Plus (Shimadzu, Kyoto, Japan). A PE capped CERI L-column 2 ODS (150 mm × 2.1 mm I. D., particle size 3 μm, Chemicals Evaluation and Research Institute, Tokyo, Japan) was used for RP-IP-LC. The conditions were as follows: mobile phase, 10 mM tributylamine and 15 mM acetic acid in water (A) and methanol (B); flow rate, 0.3 ml/min; gradient curve, 0% B at 0–1 min, 0–15% B at 1–2 min, 15-50% B at 4–9 min, 50–55% B at 9–11.5 min, 55–100% B at 11.5–12 min and 100-0% B at 13–13.5 min and 0% B at 13.5–18 min; injection volume, 3 μl; and column oven temperature, 45 °C. The mode of mass analysis was negative ion mode. The probe position was +1.5 mm, the desolvation line temperature was 250 °C, the nebulizer gas flow was 2 l/min, the drying gas flow was 15 l/min, and the heat block temperature was 400 °C. The other MS parameters were determined by auto-tuning. The scheduled multiple reaction monitoring (MRM) mode was applied throughout the analysis. The peaks of isotopomers of each target metabolite were identified by comparison of the shapes and retention times with that of the authentic compounds, and those areas were determined by LabSolutions version 5.60 (Shimadzu). The LC/QqQ–MS parameters of metabolites and details of MRM method are shown in supplementary Table S4. The data was obtained from three independent cultivations.

## Additional Information

**How to cite this article**: Shimizu, R. *et al*. New Insight into the Role of the Calvin Cycle: Reutilization of CO_2_ Emitted through Sugar Degradation. *Sci. Rep*. **5**, 11617; doi: 10.1038/srep11617 (2015).

## Supplementary Material

Supplementary Information

## Figures and Tables

**Table 1:** Production of and ^13^C abundance in P(3HB) on [1-^13^C_1_]-glucose, and carbon yield of P(3HB) on glucose with *R. eutropha* H16G and CBB cycle-inactivated strains.

*R. eutropha* strain	Time (h)	P(3HB)^a^ (g/L)	^13^C abundance in P(3HB)^b^ (%)	P(3HB) yield^c^ (g/g-glucose)
H16G	2	0.21 ± 0.21	2.09 ± 0.00	0.345
	12	1.61 ± 0.06	5.64 ± 0.00	
H16G∆*cbbR*	2	0.20 ± 0.00	1.79 ± 0.00	0.294
	12	1.38 ± 0.03	2.34 ± 0.00	
H16G∆∆*cbbLS*	2	0.22 ± 0.04	1.68 ± 0.08	0.286
	12	1.00 ± 0.02	1.91 ± 0.02	

^*a*^P(3HB) produced from [1-^13^C_1_]-glucose by 2-step cultivation.

^*b*^Means of ^13^C/^12^C ratio calculated from isotopomer abundances of the two fragments (*m/z* 45 and 87) derived from 3HB methyl ester.

^*c*^Determined from relationship between P(3HB) production and glucose consumption obtained by 6 ~ 8 independent 2-step cultivations for various incubation periods.
