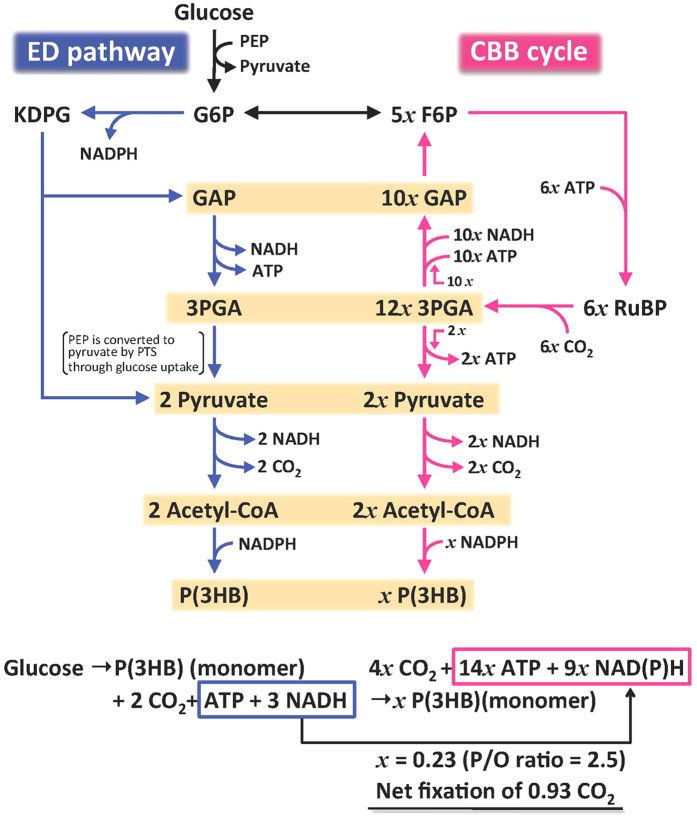# Corrigendum: New Insight into the Role of the Calvin Cycle: Reutilization of CO_2_ Emitted through Sugar Degradation

**DOI:** 10.1038/srep27961

**Published:** 2016-06-20

**Authors:** Rie Shimizu, Yudai Dempo, Yasumune Nakayama, Satoshi Nakamura, Takeshi Bamba, Eiichiro Fukusaki, Toshiaki Fukui

Scientific Reports 5: Article number: 1161710.1038/srep11617; published online: 07012015; updated: 06202016

In this Article, there is an error in Figure 3 where the formation of the ATP molecule between the reaction steps 3PGA and 2 Pyruvate should be omitted. The correct Figure 3 appears below as Figure 1.

## Figures and Tables

**Figure 1:**