# Supplementary material for: New Insight into the Role of the Calvin Cycle: Reutilization of CO2 Emitted through Sugar Degradation
Source: Sci Rep. 2015 Jul 1;5:11617. doi: 10.1038/srep11617 (PMC4486974; doi:10.1038/srep11617)
Supplement: Supplementary Information [file srep11617-s1.pdf]

Supplementary Information

New insight into the Role of the Calvin cycle: Reutilization of CO_2 Emitted through Sugar Degradation

Rie Shimizu [1], Yudai Dempo[2], Yasumune Nakayama[2], Satoshi Nakamura[1],

Takeshi Bamba[2], Eiichiro Fukusaki[2], and Toshiaki Fukui[1*]

[1]*Department of Bioengineering, Graduate School of Bioscience and Biotechnology, Tokyo Institute of Technology, 4259 Nagatsuta, Midori-ku, Yokohama 226-8501, Japan*
[2]*Department of Biotechnology, Graduate School of Engineering, Osaka University, 2-1 Yamadaoka, Suita, Osaka 565-0871, Japan*

*Corresponding author
Mailing address: tfukui@bio.titech.ac.jp

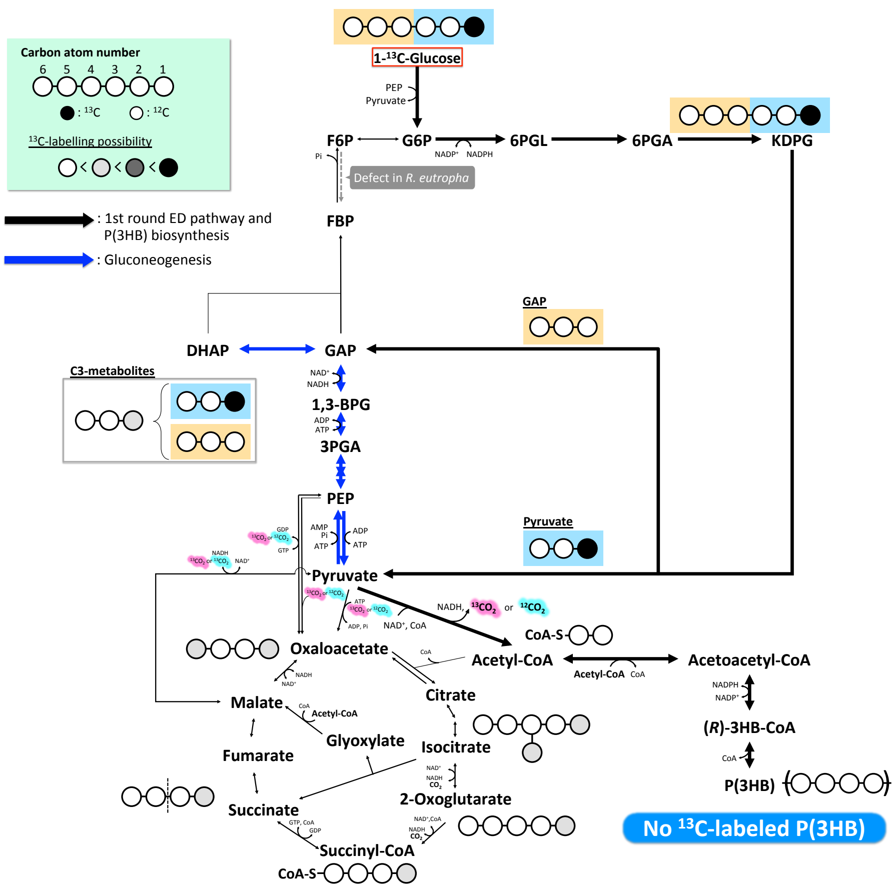

Fig. S1. Distribution of ^{13}C derived from 1-$^{13}C_1$-glucose *via* ED pathway, anaplerosis, and TCA cycles in *R. eutropha*

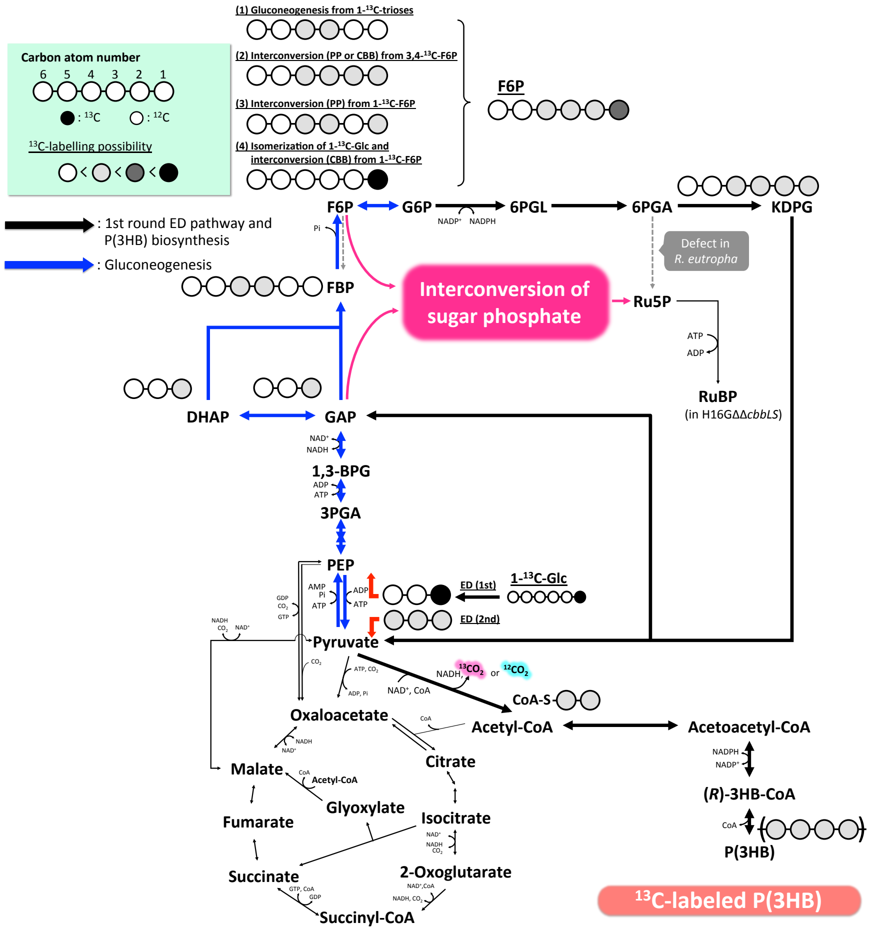

Fig. S2. Distribution of ^{13}C derived from [1-$^{13}C_1$]-glucose *via* ED pathway in combination with gluconeogenesis and interconversion of sugar phosphates in PP and CBB cycles in *R. eutropha* H16GΔ*cbbR* and H16GΔΔ*cbbLS*

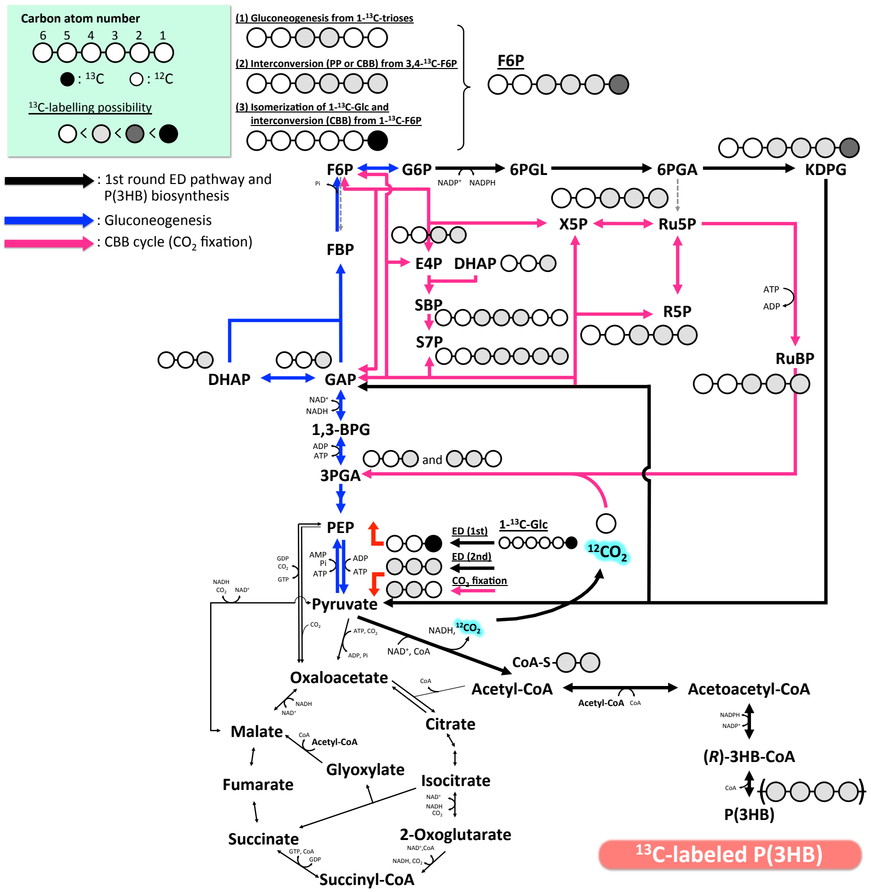

Fig. S3. Distribution of ^{13}C derived from [1-$^{13}C_1$]-glucose *via* ED pathway in combination with gluconeogenesis, interconversion of sugar phosphates in CBB cycle and $^{12}CO_2$ fixation in *R. eutropha* H16G

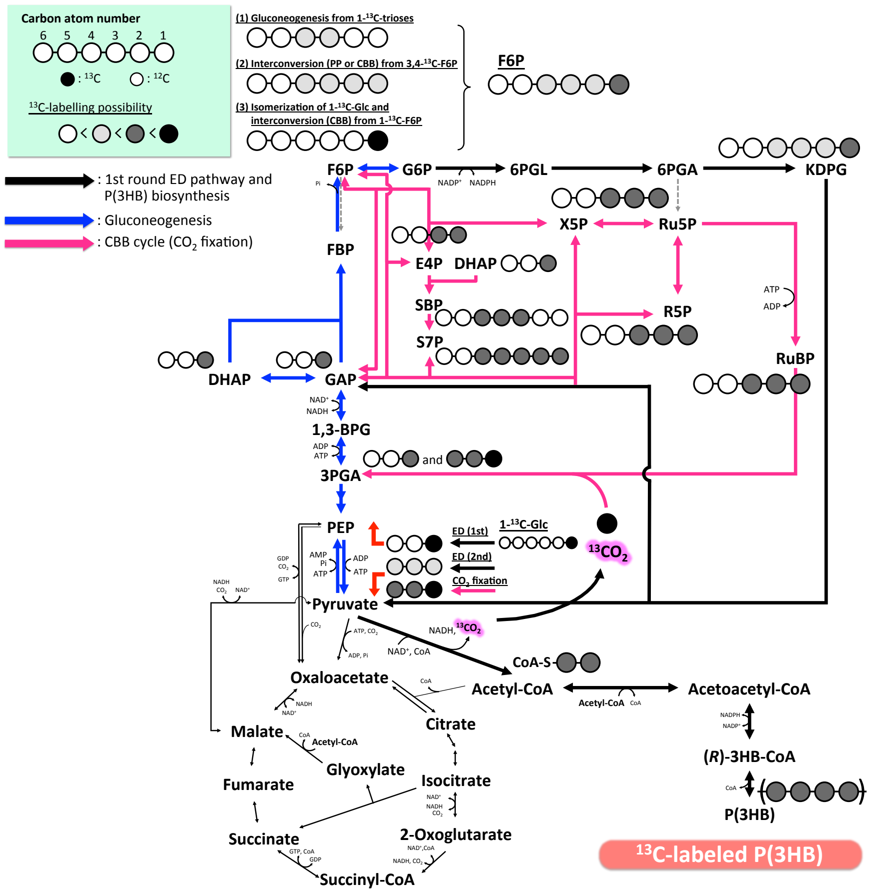

Fig. S4. Distribution of ^{13}C derived from [1-$^{13}C_1$]-glucose *via* ED pathway in combination with gluconeogenesis, interconversion of sugar phosphates in CBB cycle and $^{13}CO_2$ fixation in *R. eutropha* H16G

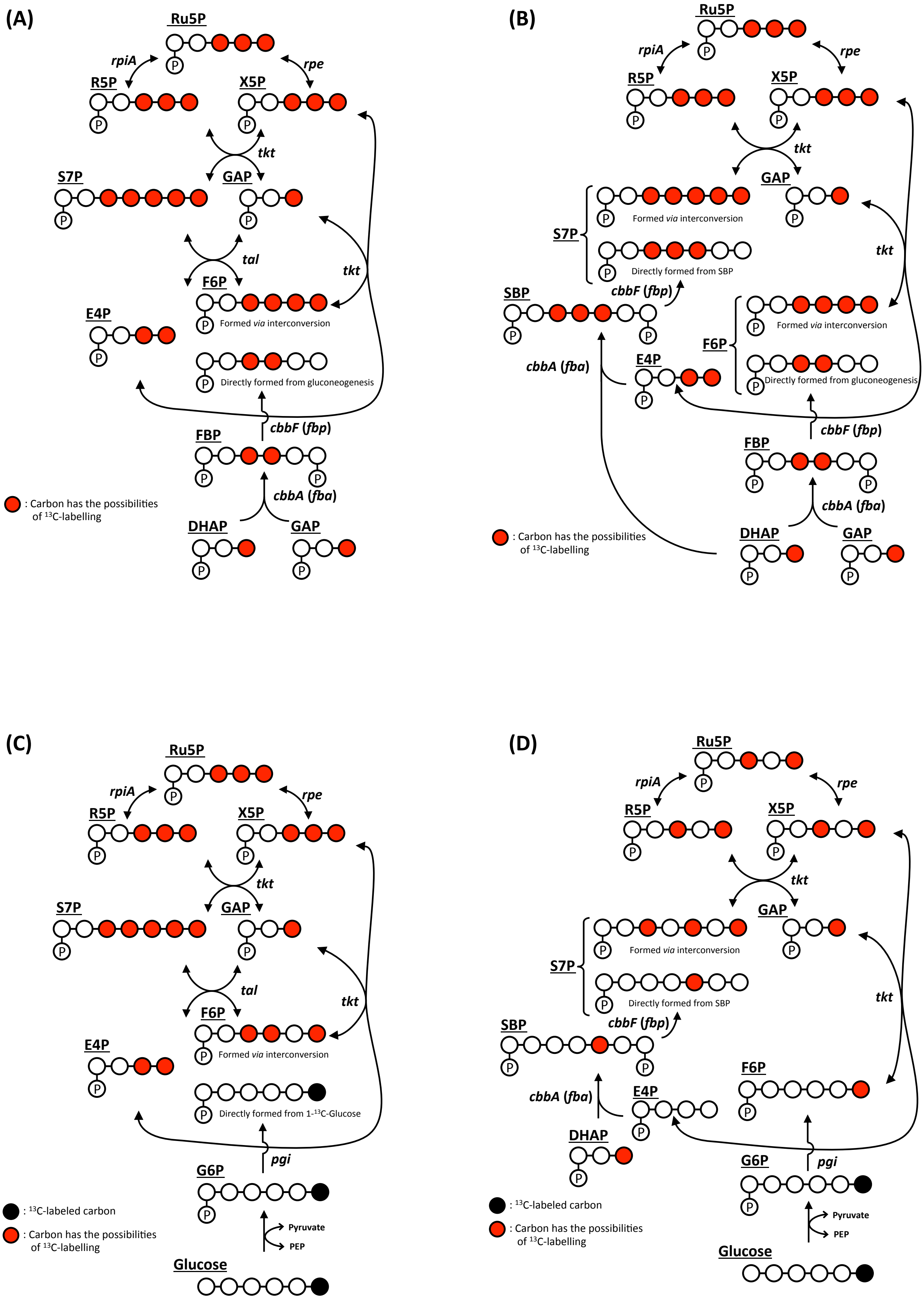

Fig. S5. Details of possible distribution of ^{13}C derived from [1-$^{13}C_1$]-triose phosphates *via* gluconeogenesis and interconversion of sugar phosphate in PP pathway (A) and CBB cycle (B), and that of ^{13}C derived from [1-$^{13}C_1$]-glucose *via* interconversion of sugar phosphates in PP pathway (C) and CBB cycle (D).

Table S1. Abbreviation of metabolites in this study.

Name	Abbreviation
(*R*)-3-Hydroxybutyryl-CoA	(*R*)-3HB-CoA
1,3-Bisphosphoglycerate	1,3-BPG
2-Keto-3-deoxygluconate-6-phosphate	KDPG
2-Oxoglutarate	2-OG
2-Phosphoglycerate	2PGA
3-Phosphoglycerate	3PGA
6-Phospho-1,5-gluconolactone	6PGL
6-Phosphogluconate	6PGA
α-Glycerophosphate	α-GP
Aspartate	Asp
Coenzyme A	CoA
Dihydroxyacetonephosphate	DHAP
Erythrose-4-phosphate	E4P
Fructose-1,6-bisphosphate	FBP
Fructose-6-phosphate	F6P
Glucose-6-phodphate	G6P
Glutamate	Glu
Glyeraldehyde-3-phosphate	GAP
Isoleucine	Ile
Leucine	Leu
Methionine	Met
Phenylalanine	Phe
Phosphoenol pyruvate	PEP
Phosphoribosyl pyrophosphate	PRPP
Poly((*R*)-3-hydroxybutyrate)	P(3HB)
Ribose-5-phosphate	R5P
Ribulose-1,5-bisphosphate	RuBP
Ribulose-5-phosphate	Ru5P
Sedoheptulose-1,7-bisphosphate	SBP
Sedoheptulose-7-phosphate	S7P
Serine	Ser
Threonine	Thr
Tyrosine	Tyr

Table S2. Abundances of isotopomers in each metabolites in *R. eutropha* H16G and the CBB cycle-inactivated strains incubated on [1-$^{13}C_1$]-glucose.

		H16G		H16GΔ*cbbR*		H16GΔΔ*cbbLS*	
Metabolite	Nomber of ^{13}C (i)	2 h	12 h	2 h	12 h	2 h	12 h
2-OG	0	$7.91\times10^{-1} \pm 1.80\times10^{-2}$	$6.40\times10^{-1} \pm 3.07\times10^{-2}$	$8.65\times10^{-1} \pm 1.22\times10^{-2}$	$8.22\times10^{-1} \pm 3.90\times10^{-2}$	$9.12\times10^{-1} \pm 5.64\times10^{-2}$	$8.69\times10^{-1} \pm 1.71\times10^{-2}$
2-OG	1	$1.87\times10^{-1} \pm 2.08\times10^{-2}$	$2.90\times10^{-1} \pm 1.82\times10^{-2}$	$1.29\times10^{-1} \pm 1.51\times10^{-2}$	$1.66\times10^{-1} \pm 3.14\times10^{-2}$	$8.79\times10^{-2} \pm 5.64\times10^{-2}$	$1.31\times10^{-1} \pm 1.71\times10^{-2}$
2-OG	2	$2.23\times10^{-2} \pm 8.85\times10^{-3}$	$6.99\times10^{-2} \pm 1.91\times10^{-2}$	$5.94\times10^{-3} \pm 7.20\times10^{-3}$	$1.21\times10^{-2} \pm 1.05\times10^{-2}$	0 ± 0	0 ± 0
2-OG	>3	0 ± 0	0 ± 0	0 ± 0	0 ± 0	0 ± 0	0 ± 0
3PGA	0	$8.91\times10^{-1} \pm 6.59\times10^{-3}$	$8.88\times10^{-1} \pm 7.13\times10^{-3}$	$9.23\times10^{-1} \pm 4.83\times10^{-3}$	$9.23\times10^{-1} \pm 2.83\times10^{-3}$	$9.36\times10^{-1} \pm 1.72\times10^{-3}$	$9.43\times10^{-1} \pm 1.06\times10^{-3}$
3PGA	1	$8.20\times10^{-2} \pm 9.73\times10^{-3}$	$9.01\times10^{-2} \pm 6.57\times10^{-3}$	$6.18\times10^{-2} \pm 7.09\times10^{-3}$	$5.92\times10^{-2} \pm 1.27\times10^{-3}$	$4.79\times10^{-2} \pm 2.65\times10^{-4}$	$4.60\times10^{-2} \pm 2.03\times10^{-4}$
3PGA	2	$2.50\times10^{-2} \pm 4.12\times10^{-3}$	$2.05\times10^{-2} \pm 3.35\times10^{-4}$	$1.45\times10^{-2} \pm 2.20\times10^{-3}$	$1.66\times10^{-2} \pm 1.28\times10^{-3}$	$1.53\times10^{-2} \pm 1.90\times10^{-3}$	$1.02\times10^{-2} \pm 9.52\times10^{-4}$
3PGA	3	$2.01\times10^{-3} \pm 1.62\times10^{-3}$	$1.22\times10^{-3} \pm 4.92\times10^{-4}$	$9.14\times10^{-4} \pm 3.95\times10^{-4}$	$7.30\times10^{-4} \pm 7.91\times10^{-4}$	$1.00\times10^{-3} \pm 1.83\times10^{-4}$	$4.33\times10^{-4} \pm 7.34\times10^{-5}$
α-GP	0	$8.95\times10^{-1} \pm 1.04\times10^{-2}$	$8.87\times10^{-1} \pm 1.80\times10^{-2}$	$9.32\times10^{-1} \pm 2.13\times10^{-3}$	$9.29\times10^{-1} \pm 1.50\times10^{-2}$	$9.45\times10^{-1} \pm 3.86\times10^{-3}$	$9.46\times10^{-1} \pm 7.92\times10^{-3}$
α-GP	1	$1.05\times10^{-1} \pm 1.04\times10^{-2}$	$1.13\times10^{-1} \pm 1.80\times10^{-2}$	$6.83\times10^{-2} \pm 2.13\times10^{-3}$	$7.09\times10^{-2} \pm 1.50\times10^{-2}$	$5.54\times10^{-2} \pm 3.86\times10^{-3}$	$5.44\times10^{-2} \pm 7.92\times10^{-3}$
α-GP	>2	0 ± 0	0 ± 0	0 ± 0	0 ± 0	0 ± 0	0 ± 0
Asp	0	$7.64\times10^{-1} \pm 4.13\times10^{-2}$	$7.01\times10^{-1} \pm 5.62\times10^{-2}$	$7.79\times10^{-1} \pm 5.11\times10^{-2}$	$6.66\times10^{-1} \pm 3.13\times10^{-2}$	$9.17\times10^{-1} \pm 1.02\times10^{-2}$	$8.38\times10^{-1} \pm 2.83\times10^{-2}$
Asp	1	$2.08\times10^{-1} \pm 3.73\times10^{-2}$	$2.40\times10^{-1} \pm 3.42\times10^{-2}$	$2.03\times10^{-1} \pm 4.37\times10^{-2}$	$2.82\times10^{-1} \pm 3.77\times10^{-2}$	$7.34\times10^{-2} \pm 1.05\times10^{-2}$	$1.41\times10^{-1} \pm 2.00\times10^{-2}$
Asp	2	$2.76\times10^{-2} \pm 1.01\times10^{-2}$	$5.95\times10^{-2} \pm 2.20\times10^{-2}$	$1.80\times10^{-2} \pm 1.43\times10^{-2}$	$5.22\times10^{-2} \pm 1.75\times10^{-2}$	$9.59\times10^{-3} \pm 3.45\times10^{-3}$	$2.08\times10^{-2} \pm 9.28\times10^{-3}$
Asp	>3	0 ± 0	0 ± 0	0 ± 0	0 ± 0	0 ± 0	0 ± 0
1,3-BPG	0	$8.65\times10^{-1} \pm 1.85\times10^{-2}$	$8.33\times10^{-1} \pm 2.57\times10^{-2}$	$9.04\times10^{-1} \pm 2.46\times10^{-2}$	$9.23\times10^{-1} \pm 4.96\times10^{-3}$	$9.42\times10^{-1} \pm 1.95\times10^{-3}$	$9.49\times10^{-1} \pm 3.71\times10^{-3}$
1,3-BPG	1	$1.05\times10^{-1} \pm 8.82\times10^{-3}$	$1.27\times10^{-1} \pm 1.45\times10^{-2}$	$6.91\times10^{-2} \pm 1.55\times10^{-2}$	$5.76\times10^{-2} \pm 5.09\times10^{-3}$	$4.44\times10^{-2} \pm 3.43\times10^{-3}$	$4.01\times10^{-2} \pm 2.65\times10^{-3}$
1,3-BPG	2	$2.77\times10^{-2} \pm 7.30\times10^{-3}$	$3.72\times10^{-2} \pm 1.04\times10^{-2}$	$2.36\times10^{-2} \pm 9.31\times10^{-3}$	$1.87\times10^{-2} \pm 1.07\times10^{-3}$	$1.36\times10^{-2} \pm 1.49\times10^{-3}$	$1.14\times10^{-2} \pm 1.06\times10^{-3}$
1,3-BPG	3	$2.43\times10^{-3} \pm 2.97\times10^{-3}$	$2.69\times10^{-3} \pm 8.69\times10^{-4}$	$3.48\times10^{-3} \pm 2.36\times10^{-3}$	$9.24\times10^{-4} \pm 6.60\times10^{-4}$	0 ± 0	0 ± 0
CoA	0	$6.60\times10^{-1} \pm 4.58\times10^{-2}$	$4.83\times10^{-1} \pm 2.55\times10^{-2}$	$6.83\times10^{-1} \pm 1.65\times10^{-3}$	$5.83\times10^{-1} \pm 8.41\times10^{-3}$	$6.77\times10^{-1} \pm 1.00\times10^{-2}$	$5.99\times10^{-1} \pm 6.59\times10^{-3}$
CoA	1	$2.33\times10^{-1} \pm 3.61\times10^{-2}$	$2.94\times10^{-1} \pm 7.82\times10^{-3}$	$2.08\times10^{-1} \pm 2.11\times10^{-3}$	$2.77\times10^{-1} \pm 1.14\times10^{-2}$	$2.18\times10^{-1} \pm 8.64\times10^{-3}$	$2.59\times10^{-1} \pm 7.09\times10^{-3}$
CoA	2	$8.19\times10^{-2} \pm 8.13\times10^{-3}$	$1.37\times10^{-1} \pm 2.33\times10^{-2}$	$8.66\times10^{-2} \pm 5.56\times10^{-3}$	$1.08\times10^{-1} \pm 2.06\times10^{-3}$	$8.19\times10^{-2} \pm 2.38\times10^{-3}$	$1.07\times10^{-1} \pm 9.88\times10^{-3}$
CoA	3	$1.92\times10^{-2} \pm 1.55\times10^{-3}$	$6.99\times10^{-2} \pm 1.91\times10^{-2}$	$1.95\times10^{-2} \pm 2.56\times10^{-3}$	$2.35\times10^{-2} \pm 6.77\times10^{-3}$	$1.95\times10^{-2} \pm 1.54\times10^{-3}$	$2.81\times10^{-2} \pm 2.03\times10^{-3}$
CoA	4	$5.42\times10^{-3} \pm 1.09\times10^{-3}$	$1.66\times10^{-2} \pm 4.92\times10^{-3}$	$3.32\times10^{-3} \pm 4.08\times10^{-4}$	$8.28\times10^{-3} \pm 7.17\times10^{-3}$	$3.17\times10^{-3} \pm 7.35\times10^{-4}$	$5.53\times10^{-3} \pm 3.52\times10^{-3}$
CoA	5	0 ± 0	0 ± 0	0 ± 0	0 ± 0	$5.71\times10^{-5} \pm 9.89\times10^{-5}$	$1.64\times10^{-3} \pm 3.86\times10^{-4}$
DHAP	0	$9.00\times10^{-1} \pm 3.12\times10^{-3}$	$9.04\times10^{-1} \pm 7.97\times10^{-4}$	$9.20\times10^{-1} \pm 1.07\times10^{-2}$	$9.27\times10^{-1} \pm 4.71\times10^{-3}$	$9.47\times10^{-1} \pm 1.79\times10^{-3}$	$9.49\times10^{-1} \pm 9.13\times10^{-4}$
DHAP	1	$8.45\times10^{-2} \pm 2.97\times10^{-3}$	$7.90\times10^{-2} \pm 9.38\times10^{-4}$	$6.78\times10^{-2} \pm 9.51\times10^{-3}$	$6.06\times10^{-2} \pm 3.31\times10^{-3}$	$4.49\times10^{-2} \pm 1.81\times10^{-3}$	$4.22\times10^{-2} \pm 1.78\times10^{-4}$
DHAP	2	$1.50\times10^{-2} \pm 6.70\times10^{-4}$	$1.66\times10^{-2} \pm 6.89\times10^{-4}$	$1.15\times10^{-2} \pm 1.28\times10^{-3}$	$1.20\times10^{-2} \pm 1.44\times10^{-3}$	$7.97\times10^{-3} \pm 2.44\times10^{-4}$	$8.63\times10^{-3} \pm 8.40\times10^{-4}$
DHAP	3	$3.66\times10^{-4} \pm 1.88\times10^{-4}$	$6.01\times10^{-4} \pm 1.29\times10^{-4}$	$2.66\times10^{-4} \pm 5.44\times10^{-5}$	$1.60\times10^{-4} \pm 1.17\times10^{-5}$	0 ± 0	0 ± 0

Table S2. Continued.

		H16G		H16GΔ*cbbR*		H16GΔΔ*cbbLS*	
Metabolite	Nomber of ^{13}C (i)	2 h	12 h	2 h	12 h	2 h	12 h
FBP	0	$5.91\times10^{-1} \pm 2.07\times10^{-2}$	$5.07\times10^{-1} \pm 1.65\times10^{-2}$	$6.58\times10^{-1} \pm 4.12\times10^{-2}$	$6.56\times10^{-1} \pm 3.12\times10^{-2}$	$8.24\times10^{-1} \pm 1.29\times10^{-2}$	$8.06\times10^{-1} \pm 8.63\times10^{-3}$
FBP	1	$2.05\times10^{-1} \pm 1.96\times10^{-2}$	$2.48\times10^{-1} \pm 1.68\times10^{-2}$	$1.96\times10^{-1} \pm 1.75\times10^{-2}$	$1.90\times10^{-1} \pm 4.96\times10^{-3}$	$1.13\times10^{-1} \pm 5.93\times10^{-3}$	$1.17\times10^{-1} \pm 1.16\times10^{-2}$
FBP	2	$1.41\times10^{-1} \pm 3.10\times10^{-2}$	$1.35\times10^{-1} \pm 2.10\times10^{-2}$	$9.82\times10^{-2} \pm 2.09\times10^{-2}$	$9.91\times10^{-2} \pm 2.32\times10^{-2}$	$4.15\times10^{-2} \pm 1.96\times10^{-3}$	$5.11\times10^{-2} \pm 4.99\times10^{-3}$
FBP	3	$6.37\times10^{-2} \pm 1.98\times10^{-2}$	$1.10\times10^{-1} \pm 1.35\times10^{-2}$	$4.82\times10^{-2} \pm 8.96\times10^{-3}$	$5.52\times10^{-2} \pm 1.18\times10^{-2}$	$2.08\times10^{-2} \pm 1.12\times10^{-2}$	$2.60\times10^{-2} \pm 5.91\times10^{-3}$
FBP	>4	0 ± 0	0 ± 0	0 ± 0	0 ± 0	0 ± 0	0 ± 0
Fumarate	0	$7.37\times10^{-1} \pm 2.41\times10^{-2}$	$6.15\times10^{-1} \pm 3.95\times10^{-3}$	$7.86\times10^{-1} \pm 5.97\times10^{-2}$	$7.21\times10^{-1} \pm 4.58\times10^{-2}$	$8.29\times10^{-1} \pm 1.41\times10^{-2}$	$7.81\times10^{-1} \pm 4.85\times10^{-3}$
Fumarate	1	$1.88\times10^{-1} \pm 2.59\times10^{-2}$	$3.14\times10^{-1} \pm 1.10\times10^{-2}$	$1.68\times10^{-1} \pm 4.01\times10^{-2}$	$2.27\times10^{-1} \pm 3.04\times10^{-2}$	$1.48\times10^{-1} \pm 9.21\times10^{-3}$	$1.95\times10^{-1} \pm 7.34\times10^{-3}$
Fumarate	2	$7.53\times10^{-2} \pm 2.64\times10^{-2}$	$7.07\times10^{-2} \pm 7.02\times10^{-3}$	$4.59\times10^{-2} \pm 2.25\times10^{-2}$	$5.20\times10^{-2} \pm 1.56\times10^{-2}$	$2.28\times10^{-2} \pm 9.65\times10^{-3}$	$2.41\times10^{-2} \pm 3.44\times10^{-3}$
Fumarate	>3	0 ± 0	0 ± 0	0 ± 0	0 ± 0	0 ± 0	0 ± 0
Glu	0	$8.62\times10^{-1} \pm 1.47\times10^{-2}$	$6.60\times10^{-1} \pm 2.09\times10^{-2}$	$8.99\times10^{-1} \pm 2.59\times10^{-2}$	$8.08\times10^{-1} \pm 1.73\times10^{-3}$	$8.89\times10^{-1} \pm 1.15\times10^{-2}$	$8.71\times10^{-1} \pm 2.63\times10^{-2}$
Glu	1	$1.19\times10^{-1} \pm 2.02\times10^{-2}$	$2.64\times10^{-1} \pm 1.94\times10^{-2}$	$9.08\times10^{-2} \pm 2.35\times10^{-2}$	$1.69\times10^{-1} \pm 2.73\times10^{-3}$	$9.68\times10^{-2} \pm 3.64\times10^{-3}$	$1.21\times10^{-1} \pm 2.45\times10^{-2}$
Glu	2	$1.83\times10^{-2} \pm 7.00\times10^{-3}$	$7.61\times10^{-2} \pm 1.06\times10^{-2}$	$1.05\times10^{-2} \pm 3.36\times10^{-3}$	$2.28\times10^{-2} \pm 4.39\times10^{-3}$	$1.46\times10^{-2} \pm 1.16\times10^{-2}$	$7.99\times10^{-3} \pm 7.12\times10^{-3}$
Glu	>3	0 ± 0	0 ± 0	0 ± 0	0 ± 0	0 ± 0	0 ± 0
Isocitrate	0	$7.69\times10^{-1} \pm 1.82\times10^{-2}$	$6.45\times10^{-1} \pm 4.37\times10^{-2}$	$8.07\times10^{-1} \pm 2.69\times10^{-2}$	$7.85\times10^{-1} \pm 4.70\times10^{-2}$	$7.81\times10^{-1} \pm 1.33\times10^{-2}$	$7.33\times10^{-1} \pm 3.56\times10^{-2}$
Isocitrate	1	$1.88\times10^{-1} \pm 1.49\times10^{-2}$	$2.66\times10^{-1} \pm 2.95\times10^{-2}$	$1.66\times10^{-1} \pm 2.22\times10^{-2}$	$1.78\times10^{-1} \pm 3.41\times10^{-2}$	$1.93\times10^{-1} \pm 1.05\times10^{-2}$	$2.32\times10^{-1} \pm 3.09\times10^{-2}$
Isocitrate	2	$3.99\times10^{-2} \pm 3.11\times10^{-3}$	$7.57\times10^{-2} \pm 1.32\times10^{-2}$	$2.59\times10^{-2} \pm 4.74\times10^{-3}$	$3.49\times10^{-2} \pm 1.19\times10^{-2}$	$2.62\times10^{-2} \pm 7.03\times10^{-3}$	$3.46\times10^{-2} \pm 5.05\times10^{-3}$
Isocitrate	3	$3.40\times10^{-3} \pm 1.42\times10^{-3}$	$1.30\times10^{-2} \pm 1.12\times10^{-3}$	$8.38\times10^{-4} \pm 5.57\times10^{-4}$	$1.70\times10^{-3} \pm 1.23\times10^{-3}$	0 ± 0	0 ± 0
Isocitrate	>4	0 ± 0	0 ± 0	0 ± 0	0 ± 0	0 ± 0	0 ± 0
Ile	0	$8.39\times10^{-1} \pm 1.92\times10^{-2}$	$8.70\times10^{-1} \pm 3.92\times10^{-2}$	$8.87\times10^{-1} \pm 2.70\times10^{-2}$	$8.70\times10^{-1} \pm 4.03\times10^{-2}$	$9.17\times10^{-1} \pm 4.23\times10^{-3}$	$9.14\times10^{-1} \pm 1.81\times10^{-3}$
Ile	1	$1.61\times10^{-1} \pm 1.92\times10^{-2}$	$1.30\times10^{-1} \pm 3.92\times10^{-2}$	$1.13\times10^{-1} \pm 2.70\times10^{-2}$	$1.30\times10^{-1} \pm 4.03\times10^{-2}$	$7.25\times10^{-2} \pm 3.27\times10^{-3}$	$7.45\times10^{-2} \pm 4.81\times10^{-3}$
Ile	2	0 ± 0	0 ± 0	0 ± 0	0 ± 0	$1.08\times10^{-2} \pm 1.05\times10^{-3}$	$1.19\times10^{-2} \pm 3.00\times10^{-3}$
Ile	>3	0 ± 0	0 ± 0	0 ± 0	0 ± 0	0 ± 0	0 ± 0
Leu	0	$8.88\times10^{-1} \pm 1.82\times10^{-3}$	$8.35\times10^{-1} \pm 2.17\times10^{-2}$	$8.86\times10^{-1} \pm 2.90\times10^{-2}$	$8.84\times10^{-1} \pm 1.43\times10^{-3}$	$9.08\times10^{-1} \pm 1.99\times10^{-3}$	$8.96\times10^{-1} \pm 1.81\times10^{-2}$
Leu	1	$1.02\times10^{-1} \pm 2.51\times10^{-3}$	$1.22\times10^{-1} \pm 1.62\times10^{-2}$	$1.07\times10^{-1} \pm 2.77\times10^{-2}$	$1.02\times10^{-1} \pm 3.56\times10^{-3}$	$7.86\times10^{-2} \pm 3.86\times10^{-3}$	$9.07\times10^{-2} \pm 1.54\times10^{-2}$
Leu	2	$9.64\times10^{-3} \pm 1.66\times10^{-3}$	$2.11\times10^{-2} \pm 2.69\times10^{-3}$	$7.80\times10^{-3} \pm 1.48\times10^{-3}$	$1.42\times10^{-2} \pm 2.48\times10^{-3}$	$1.36\times10^{-2} \pm 2.45\times10^{-3}$	$1.34\times10^{-2} \pm 2.66\times10^{-3}$
Leu	3	0 ± 0	$2.21\times10^{-2} \pm 3.55\times10^{-3}$	0 ± 0	0 ± 0	0 ± 0	0 ± 0
Leu	>4	0 ± 0	0 ± 0	0 ± 0	0 ± 0	0 ± 0	0 ± 0
Malate	0	$7.79\times10^{-1} \pm 1.09\times10^{-2}$	$6.66\times10^{-1} \pm 1.03\times10^{-1}$	$8.11\times10^{-1} \pm 1.74\times10^{-2}$	$7.24\times10^{-1} \pm 1.69\times10^{-2}$	$8.43\times10^{-1} \pm 7.41\times10^{-3}$	$8.29\times10^{-1} \pm 9.90\times10^{-3}$
Malate	1	$1.80\times10^{-1} \pm 9.36\times10^{-3}$	$2.68\times10^{-1} \pm 8.32\times10^{-2}$	$1.59\times10^{-1} \pm 1.57\times10^{-2}$	$2.22\times10^{-1} \pm 1.35\times10^{-2}$	$1.39\times10^{-1} \pm 6.38\times10^{-3}$	$1.53\times10^{-1} \pm 8.28\times10^{-3}$
Malate	2	$3.65\times10^{-2} \pm 8.14\times10^{-4}$	$5.93\times10^{-2} \pm 1.93\times10^{-2}$	$2.78\times10^{-2} \pm 2.10\times10^{-3}$	$5.04\times10^{-2} \pm 3.64\times10^{-3}$	$1.64\times10^{-2} \pm 9.60\times10^{-4}$	$1.63\times10^{-2} \pm 1.52\times10^{-3}$
Malate	3	$3.69\times10^{-3} \pm 8.35\times10^{-4}$	$6.39\times10^{-3} \pm 1.57\times10^{-3}$	$2.32\times10^{-3} \pm 2.46\times10^{-4}$	$3.70\times10^{-3} \pm 3.88\times10^{-4}$	$1.40\times10^{-3} \pm 4.88\times10^{-4}$	$1.79\times10^{-3} \pm 3.06\times10^{-4}$
Malate	4	$6.09\times10^{-4} \pm 1.54\times10^{-4}$	$6.43\times10^{-4} \pm 1.24\times10^{-4}$	$3.36\times10^{-4} \pm 1.10\times10^{-4}$	$3.31\times10^{-4} \pm 3.03\times10^{-4}$	$5.27\times10^{-5} \pm 3.39\times10^{-5}$	$2.76\times10^{-5} \pm 2.14\times10^{-5}$

Table S2. Continued.

Metabolite	Nomber of ^{13}C (i)	H16G 2 h	H16G 12 h	H16GΔ*cbbR* 2 h	H16GΔ*cbbR* 12 h	H16GΔΔ*cbbLS* 2 h	H16GΔΔ*cbbLS* 12 h
Met	0	$8.40\times10^{-1} \pm 2.01\times10^{-2}$	$7.90\times10^{-1} \pm 5.88\times10^{-3}$	$8.69\times10^{-1} \pm 2.43\times10^{-2}$	$8.74\times10^{-1} \pm 2.38\times10^{-2}$	$9.02\times10^{-1} \pm 4.03\times10^{-3}$	$9.10\times10^{-1} \pm 2.67\times10^{-3}$
Met	1	$1.42\times10^{-1} \pm 1.67\times10^{-2}$	$1.67\times10^{-1} \pm 9.92\times10^{-3}$	$1.20\times10^{-1} \pm 1.90\times10^{-2}$	$1.10\times10^{-1} \pm 1.81\times10^{-2}$	$8.91\times10^{-2} \pm 9.80\times10^{-4}$	$8.12\times10^{-2} \pm 6.02\times10^{-3}$
Met	2	$1.78\times10^{-2} \pm 3.38\times10^{-3}$	$4.36\times10^{-2} \pm 7.15\times10^{-3}$	$1.17\times10^{-2} \pm 6.18\times10^{-3}$	$1.57\times10^{-2} \pm 7.88\times10^{-3}$	$8.57\times10^{-3} \pm 3.87\times10^{-3}$	$9.11\times10^{-3} \pm 3.40\times10^{-3}$
Met	>3	0 ± 0	0 ± 0	0 ± 0	0 ± 0	0 ± 0	0 ± 0
PEP	0	$6.91\times10^{-1} \pm 9.41\times10^{-3}$	$6.47\times10^{-1} \pm 2.63\times10^{-2}$	$7.73\times10^{-1} \pm 5.55\times10^{-2}$	$6.95\times10^{-1} \pm 8.79\times10^{-2}$	$9.21\times10^{-1} \pm 3.29\times10^{-3}$	$9.28\times10^{-1} \pm 5.17\times10^{-3}$
PEP	1	$2.63\times10^{-1} \pm 7.28\times10^{-3}$	$2.87\times10^{-1} \pm 2.70\times10^{-2}$	$2.02\times10^{-1} \pm 6.13\times10^{-2}$	$2.77\times10^{-1} \pm 8.64\times10^{-2}$	$6.49\times10^{-2} \pm 3.65\times10^{-3}$	$5.65\times10^{-2} \pm 5.80\times10^{-3}$
PEP	2	$4.06\times10^{-2} \pm 3.82\times10^{-3}$	$6.05\times10^{-2} \pm 9.50\times10^{-3}$	$2.15\times10^{-2} \pm 4.52\times10^{-3}$	$2.59\times10^{-2} \pm 4.43\times10^{-3}$	$1.24\times10^{-2} \pm 1.33\times10^{-3}$	$1.52\times10^{-2} \pm 8.16\times10^{-4}$
PEP	3	$5.40\times10^{-3} \pm 6.78\times10^{-4}$	$5.85\times10^{-3} \pm 2.07\times10^{-3}$	$3.04\times10^{-3} \pm 2.27\times10^{-3}$	$2.15\times10^{-3} \pm 2.47\times10^{-3}$	$1.93\times10^{-3} \pm 6.24\times10^{-4}$	$6.76\times10^{-4} \pm 5.00\times10^{-4}$
Phe	0	$8.10\times10^{-1} \pm 2.09\times10^{-2}$	$8.28\times10^{-1} \pm 4.29\times10^{-2}$	$8.27\times10^{-1} \pm 1.21\times10^{-2}$	$8.26\times10^{-1} \pm 6.25\times10^{-3}$	$8.75\times10^{-1} \pm 2.51\times10^{-3}$	$8.56\times10^{-1} \pm 1.15\times10^{-2}$
Phe	1	$1.54\times10^{-1} \pm 1.29\times10^{-2}$	$1.36\times10^{-1} \pm 3.53\times10^{-2}$	$1.56\times10^{-1} \pm 8.99\times10^{-3}$	$1.57\times10^{-1} \pm 5.22\times10^{-3}$	$1.10\times10^{-1} \pm 7.59\times10^{-4}$	$1.27\times10^{-1} \pm 1.19\times10^{-2}$
Phe	2	$3.09\times10^{-2} \pm 5.52\times10^{-3}$	$2.67\times10^{-2} \pm 3.18\times10^{-3}$	$1.71\times10^{-2} \pm 3.91\times10^{-3}$	$1.63\times10^{-2} \pm 1.93\times10^{-3}$	$1.47\times10^{-2} \pm 2.01\times10^{-3}$	$1.62\times10^{-2} \pm 3.43\times10^{-3}$
Phe	3	$5.22\times10^{-3} \pm 2.79\times10^{-3}$	$9.95\times10^{-3} \pm 5.09\times10^{-3}$	0 ± 0	0 ± 0	$3.22\times10^{-4} \pm 2.84\times10^{-4}$	$6.83\times10^{-4} \pm 5.24\times10^{-4}$
Phe	>4	0 ± 0	0 ± 0	0 ± 0	0 ± 0	0 ± 0	0 ± 0
PRPP	0	$4.69\times10^{-1} \pm 1.88\times10^{-2}$	$4.44\times10^{-1} \pm 2.30\times10^{-2}$	$4.74\times10^{-1} \pm 1.03\times10^{-2}$	$3.69\times10^{-1} \pm 2.42\times10^{-2}$	$5.44\times10^{-1} \pm 1.50\times10^{-2}$	$6.55\times10^{-1} \pm 1.80\times10^{-2}$
PRPP	1	$4.32\times10^{-1} \pm 1.41\times10^{-2}$	$4.01\times10^{-1} \pm 1.62\times10^{-2}$	$4.39\times10^{-1} \pm 3.49\times10^{-3}$	$5.23\times10^{-1} \pm 2.17\times10^{-2}$	$4.08\times10^{-1} \pm 1.55\times10^{-2}$	$3.01\times10^{-1} \pm 2.20\times10^{-2}$
PRPP	2	$8.28\times10^{-2} \pm 8.09\times10^{-3}$	$1.20\times10^{-1} \pm 8.50\times10^{-3}$	$7.14\times10^{-2} \pm 4.61\times10^{-3}$	$7.88\times10^{-2} \pm 2.64\times10^{-3}$	$4.08\times10^{-2} \pm 1.26\times10^{-2}$	$3.67\times10^{-2} \pm 7.14\times10^{-3}$
PRPP	3	$1.54\times10^{-2} \pm 2.27\times10^{-3}$	$3.15\times10^{-2} \pm 8.40\times10^{-3}$	$1.44\times10^{-2} \pm 3.43\times10^{-3}$	$2.79\times10^{-2} \pm 4.45\times10^{-3}$	$7.81\times10^{-3} \pm 1.98\times10^{-3}$	$7.28\times10^{-3} \pm 3.53\times10^{-3}$
PRPP	4	$9.53\times10^{-4} \pm 9.26\times10^{-4}$	$4.02\times10^{-3} \pm 1.27\times10^{-3}$	$1.47\times10^{-3} \pm 1.28\times10^{-3}$	$8.48\times10^{-4} \pm 9.32\times10^{-4}$	0 ± 0	0 ± 0
PRPP	5	0 ± 0	0 ± 0	0 ± 0	0 ± 0	0 ± 0	0 ± 0
R5P	0	$5.06\times10^{-1} \pm 7.81\times10^{-3}$	$5.02\times10^{-1} \pm 1.19\times10^{-2}$	$4.25\times10^{-1} \pm 1.52\times10^{-2}$	$4.73\times10^{-1} \pm 2.49\times10^{-2}$	$4.33\times10^{-1} \pm 4.29\times10^{-2}$	$5.57\times10^{-1} \pm 2.34\times10^{-2}$
R5P	1	$4.20\times10^{-1} \pm 1.89\times10^{-2}$	$4.21\times10^{-1} \pm 4.00\times10^{-3}$	$4.83\times10^{-1} \pm 7.61\times10^{-3}$	$4.70\times10^{-1} \pm 2.34\times10^{-2}$	$4.98\times10^{-1} \pm 3.62\times10^{-2}$	$3.31\times10^{-1} \pm 4.55\times10^{-2}$
R5P	2	$7.47\times10^{-2} \pm 1.16\times10^{-2}$	$7.71\times10^{-2} \pm 1.39\times10^{-2}$	$9.15\times10^{-2} \pm 1.19\times10^{-2}$	$5.69\times10^{-2} \pm 1.71\times10^{-3}$	$4.70\times10^{-2} \pm 2.03\times10^{-2}$	$8.22\times10^{-2} \pm 2.02\times10^{-2}$
R5P	3	0 ± 0	0 ± 0	0 ± 0	0 ± 0	$2.26\times10^{-2} \pm 6.15\times10^{-3}$	$2.98\times10^{-2} \pm 4.05\times10^{-3}$
R5P	>4	0 ± 0	0 ± 0	0 ± 0	0 ± 0	0 ± 0	0 ± 0
Ru5P	0	$4.25\times10^{-1} \pm 1.18\times10^{-2}$	$4.31\times10^{-1} \pm 2.69\times10^{-2}$	$4.06\times10^{-1} \pm 2.04\times10^{-2}$	$4.10\times10^{-1} \pm 3.12\times10^{-2}$	$3.96\times10^{-1} \pm 2.40\times10^{-2}$	$5.67\times10^{-1} \pm 1.94\times10^{-2}$
Ru5P	1	$3.34\times10^{-1} \pm 1.57\times10^{-2}$	$4.00\times10^{-1} \pm 2.91\times10^{-2}$	$3.67\times10^{-1} \pm 3.42\times10^{-2}$	$3.78\times10^{-1} \pm 1.83\times10^{-2}$	$5.12\times10^{-1} \pm 3.21\times10^{-2}$	$3.64\times10^{-1} \pm 1.71\times10^{-2}$
Ru5P	2	$1.93\times10^{-1} \pm 1.98\times10^{-2}$	$1.30\times10^{-1} \pm 5.92\times10^{-3}$	$1.78\times10^{-1} \pm 1.39\times10^{-2}$	$1.63\times10^{-1} \pm 3.12\times10^{-2}$	$7.95\times10^{-2} \pm 6.06\times10^{-3}$	$5.74\times10^{-2} \pm 3.66\times10^{-3}$
Ru5P	3	$4.76\times10^{-2} \pm 6.15\times10^{-3}$	$3.91\times10^{-2} \pm 6.17\times10^{-3}$	$4.91\times10^{-2} \pm 1.29\times10^{-3}$	$4.88\times10^{-2} \pm 1.67\times10^{-2}$	$1.27\times10^{-2} \pm 4.72\times10^{-3}$	$1.16\times10^{-2} \pm 4.87\times10^{-4}$
Ru5P	>4	0 ± 0	0 ± 0	0 ± 0	0 ± 0	0 ± 0	0 ± 0

Table S2. Continued.

Metabolite	Number of ^{13}C (i)	H16G<br>2 h	 12 h	H16GΔ*cbbR*<br>2 h	 12 h	H16GΔΔ*cbbLS*<br>2 h	 12 h
RuBP	0	$5.29\times10^{-1} \pm 4.26\times10^{-2}$	$4.79\times10^{-1} \pm 8.72\times10^{-3}$			$5.95\times10^{-1} \pm 1.87\times10^{-2}$	$6.91\times10^{-1} \pm 3.52\times10^{-3}$
RuBP	1	$3.43\times10^{-1} \pm 3.02\times10^{-2}$	$3.92\times10^{-1} \pm 1.59\times10^{-2}$			$3.52\times10^{-1} \pm 2.37\times10^{-2}$	$2.73\times10^{-1} \pm 3.90\times10^{-3}$
RuBP	2	$1.28\times10^{-1} \pm 1.55\times10^{-2}$	$1.29\times10^{-1} \pm 7.73\times10^{-3}$	Not detected	Not detected	$3.76\times10^{-2} \pm 2.27\times10^{-3}$	$2.81\times10^{-2} \pm 1.01\times10^{-3}$
RuBP	3	0 ± 0	0 ± 0			$1.51\times10^{-2} \pm 6.33\times10^{-3}$	$7.81\times10^{-3} \pm 9.30\times10^{-4}$
RuBP	>4	0 ± 0	0 ± 0			0 ± 0	0 ± 0
S7P	0	$4.09\times10^{-1} \pm 9.81\times10^{-3}$	$3.43\times10^{-1} \pm 4.92\times10^{-3}$	$2.74\times10^{-1} \pm 3.19\times10^{-2}$	$2.14\times10^{-1} \pm 1.41\times10^{-2}$	$2.60\times10^{-1} \pm 2.43\times10^{-2}$	$4.78\times10^{-1} \pm 1.15\times10^{-2}$
S7P	1	$3.90\times10^{-1} \pm 4.70\times10^{-3}$	$4.01\times10^{-1} \pm 2.41\times10^{-3}$	$4.83\times10^{-1} \pm 4.22\times10^{-2}$	$4.77\times10^{-1} \pm 8.56\times10^{-3}$	$5.46\times10^{-1} \pm 1.44\times10^{-2}$	$3.51\times10^{-1} \pm 4.81\times10^{-3}$
S7P	2	$1.65\times10^{-1} \pm 5.12\times10^{-3}$	$1.97\times10^{-1} \pm 3.38\times10^{-3}$	$1.89\times10^{-1} \pm 1.28\times10^{-2}$	$2.47\times10^{-1} \pm 1.11\times10^{-2}$	$1.77\times10^{-1} \pm 2.52\times10^{-2}$	$1.54\times10^{-1} \pm 5.70\times10^{-3}$
S7P	3	$3.64\times10^{-2} \pm 3.93\times10^{-3}$	$5.99\times10^{-2} \pm 1.46\times10^{-3}$	$5.39\times10^{-2} \pm 4.27\times10^{-3}$	$6.23\times10^{-2} \pm 4.05\times10^{-3}$	$1.70\times10^{-2} \pm 4.05\times10^{-3}$	$1.69\times10^{-2} \pm 4.38\times10^{-3}$
S7P	>4	0 ± 0	0 ± 0	0 ± 0	0 ± 0	0 ± 0	0 ± 0
Ser	0	$8.25\times10^{-1} \pm 1.16\times10^{-2}$	$8.17\times10^{-1} \pm 6.60\times10^{-2}$	$8.42\times10^{-1} \pm 2.25\times10^{-2}$	$8.11\times10^{-1} \pm 1.47\times10^{-2}$	$8.57\times10^{-1} \pm 4.32\times10^{-2}$	$8.53\times10^{-1} \pm 2.08\times10^{-2}$
Ser	1	$8.75\times10^{-2} \pm 5.79\times10^{-3}$	$9.17\times10^{-2} \pm 3.30\times10^{-2}$	$7.91\times10^{-2} \pm 1.13\times10^{-2}$	$9.45\times10^{-2} \pm 7.33\times10^{-3}$	$7.15\times10^{-2} \pm 2.16\times10^{-2}$	$7.34\times10^{-2} \pm 1.04\times10^{-2}$
Ser	2	$8.75\times10^{-2} \pm 5.79\times10^{-3}$	$9.17\times10^{-2} \pm 3.30\times10^{-2}$	$7.91\times10^{-2} \pm 1.13\times10^{-2}$	$9.45\times10^{-2} \pm 7.33\times10^{-3}$	$7.15\times10^{-2} \pm 2.16\times10^{-2}$	$7.34\times10^{-2} \pm 1.04\times10^{-2}$
Ser	3	0 ± 0	0 ± 0	0 ± 0	0 ± 0	0 ± 0	0 ± 0
Succinate	0	$7.86\times10^{-1} \pm 4.58\times10^{-3}$	$6.54\times10^{-1} \pm 2.05\times10^{-2}$	$8.78\times10^{-1} \pm 2.72\times10^{-3}$	$8.28\times10^{-1} \pm 1.26\times10^{-2}$	$8.43\times10^{-1} \pm 5.61\times10^{-3}$	$8.21\times10^{-1} \pm 1.67\times10^{-2}$
Succinate	1	$1.90\times10^{-1} \pm 6.33\times10^{-3}$	$2.97\times10^{-1} \pm 1.93\times10^{-2}$	$1.13\times10^{-1} \pm 2.98\times10^{-3}$	$1.56\times10^{-1} \pm 1.09\times10^{-2}$	$1.45\times10^{-1} \pm 5.39\times10^{-3}$	$1.67\times10^{-1} \pm 1.59\times10^{-2}$
Succinate	2	$2.22\times10^{-2} \pm 2.09\times10^{-3}$	$4.50\times10^{-2} \pm 2.55\times10^{-3}$	$8.34\times10^{-3} \pm 7.23\times10^{-4}$	$1.61\times10^{-2} \pm 3.05\times10^{-3}$	$1.19\times10^{-2} \pm 7.47\times10^{-4}$	$1.15\times10^{-2} \pm 1.00\times10^{-3}$
Succinate	3	$1.16\times10^{-3} \pm 2.03\times10^{-4}$	$3.55\times10^{-3} \pm 2.26\times10^{-4}$	$2.67\times10^{-4} \pm 3.74\times10^{-4}$	$1.61\times10^{-4} \pm 6.86\times10^{-5}$	$4.38\times10^{-4} \pm 2.27\times10^{-4}$	$5.95\times10^{-4} \pm 1.02\times10^{-4}$
Succinate	4	0 ± 0	0 ± 0	0 ± 0	0 ± 0	0 ± 0	0 ± 0
Thr	0	$8.62\times10^{-1} \pm 6.49\times10^{-3}$	$8.29\times10^{-1} \pm 3.12\times10^{-2}$	$8.78\times10^{-1} \pm 3.18\times10^{-3}$	$8.40\times10^{-1} \pm 1.95\times10^{-2}$	$9.07\times10^{-1} \pm 5.71\times10^{-3}$	$8.87\times10^{-1} \pm 9.10\times10^{-3}$
Thr	1	$1.19\times10^{-1} \pm 3.74\times10^{-3}$	$1.41\times10^{-1} \pm 2.24\times10^{-2}$	$1.07\times10^{-1} \pm 1.78\times10^{-3}$	$1.35\times10^{-1} \pm 1.61\times10^{-2}$	$8.45\times10^{-2} \pm 6.12\times10^{-3}$	$9.47\times10^{-2} \pm 1.90\times10^{-3}$
Thr	2	$1.82\times10^{-2} \pm 6.22\times10^{-3}$	$2.99\times10^{-2} \pm 1.01\times10^{-2}$	$1.55\times10^{-2} \pm 1.83\times10^{-3}$	$2.46\times10^{-2} \pm 5.80\times10^{-3}$	$8.90\times10^{-3} \pm 8.48\times10^{-4}$	$1.87\times10^{-2} \pm 7.24\times10^{-3}$
Thr	>3	0 ± 0	0 ± 0	0 ± 0	0 ± 0	0 ± 0	0 ± 0
Tyr	0	$7.18\times10^{-1} \pm 1.25\times10^{-2}$	$7.07\times10^{-1} \pm 4.14\times10^{-2}$	$7.77\times10^{-1} \pm 1.35\times10^{-2}$	$7.44\times10^{-1} \pm 3.01\times10^{-2}$	$8.36\times10^{-1} \pm 1.43\times10^{-2}$	$8.13\times10^{-1} \pm 1.90\times10^{-2}$
Tyr	1	$2.29\times10^{-1} \pm 1.12\times10^{-2}$	$1.96\times10^{-1} \pm 1.22\times10^{-2}$	$1.87\times10^{-1} \pm 9.80\times10^{-3}$	$2.15\times10^{-1} \pm 3.14\times10^{-2}$	$1.51\times10^{-1} \pm 1.49\times10^{-2}$	$1.69\times10^{-1} \pm 2.08\times10^{-2}$
Tyr	2	$4.54\times10^{-2} \pm 5.24\times10^{-3}$	$7.07\times10^{-2} \pm 1.79\times10^{-2}$	$3.59\times10^{-2} \pm 4.36\times10^{-3}$	$4.09\times10^{-2} \pm 1.34\times10^{-3}$	$1.24\times10^{-2} \pm 3.04\times10^{-3}$	$1.83\times10^{-2} \pm 3.82\times10^{-3}$
Tyr	3	$7.60\times10^{-3} \pm 6.58\times10^{-3}$	$2.62\times10^{-2} \pm 1.23\times10^{-2}$	0 ± 0	0 ± 0	0 ± 0	0 ± 0
Tyr	>4	0 ± 0	0 ± 0	0 ± 0	0 ± 0	0 ± 0	0 ± 0

The abbreviations are shown in Table S1.

m_i represents the abundance of each isotopomer of the metabolites in which i ^{13}C atoms are incorporated, calculated by following equation:

$$m_i = \frac{M_i}{\sum_{j=0}^{n} M_j}$$

where M_i represents the peak intensity of each the isotopomer.

The results shown in grey (S7P, R5P, Isocitrate) were unreliable due to coelution of unknown contaminants.

Table S3. Primers used in qRT-PCR analysis.

Name	Sequence	Amplification size
bfr2-5'	CGCGATCAACCAGTATTTCC	228 bp
bfr2-3'	GTCTGCTCCAGCTTCAGGTC	
cbbL-F-RT-PCR	TCCACCGGCATCATCGT	218 bp
cbbL-R-RT-PCR	GCGTCCATCACGAACAGGAA	
cbbP-F-RT-PCR	GCGCACCGGCAATATGAA	218 bp
cbbP-R-RT-PCR	GGCCCTCGTAGAACAGCAGA	
cbbF-F-RT-PCR	ATGGAGGCGCCTTACCAGA	227 bp
cbbF-R-RT-PCR	ATGGTGGTGGGACCGTAGAG	

Table S4. MRM transition in this study.

Metabolite	RT[a]	MRM	CE[b]
Ser	1.14	104>74.1, 105>74.1, 105>75.1, 106>75.1, 106>76.1, 107>76.1	16
Thr	1.17	118>74.05, 119>74.05, 119>75.05, 120>74.05, 120>75.05, 120>76.05, 121>75.05, 121>76.05, 122>76.05	16
Met	1.79	148>47.05, 149>47.05, 149>48.05, 150>47.05, 150>48.05, 151>47.05, 151>48.05 152>47.05, 152>48.05, 153>48.05	14
Tyr	2.32	180>163.05, 181>164.05, 182>165.05, 183>166.05, 184>167.05 185>168.05, 186>169.05, 187>170.05, 188>171.05, 189>172.05	18
Ile	2.34	190.1>130.1, 191.1>131.1, 192.1>132.1, 193.1>133.1, 194.1>134.1, 195.1>135.1, 196.1>136.1	10
Leu	2.56	190.1>130.1, 191.1>131.1, 192.1>132.1, 193.1>133.1, 194.1>134.1, 195.1>135.1, 196.1>136.1	10
Phe	3.84	164>147, 165>148, 166>149, 167>150, 168>151, 169>152, 170>153, 171>154, 172>155, 173>156	18
Glu	3.89	146>128.05, 147>129.05, 148>130.05, 149>131.05, 150>132.05, 151>133.05	17
Asp	3.93	132>115.1, 133>116.1, 134>117.1, 135>118.1 136>119.1	16
R5P	5.16	229.05>79.05, 230.05>79.05, 231.05>79.05, 232.05>79.05, 233.05>79.05, 234.05>79.05	47
S7P	5.39	289>97, 290>97, 291>97, 292>97, 293>97, 294>97, 295>97, 296>97	23
α-GP	5.76	171.1>79.05, 172.1>79.05, 173.1>79.05, 174.1>79.05	18
Ru5P	6.49	229.05>79.05, 230.05>79.05, 231.05>79.05, 232.05>79.05, 233.05>79.05, 234.05>79.05	47
DHAP	7.20	169>97.05, 170>97.05, 171>97.05, 172>97.05	12
Succinate	8.80	117>73.1, 118>73.1, 118>74.1, 119>74.1, 119>75.1, 120>75.1, 120>76.1, 121>76.1	15
Malate	9.29	133>115.1, 134>116.1, 135>117.1, 136>118.1, 137>119.1	17
2-OG	9.59	145>101, 146>101, 146>102, 147>102, 147>103, 148>103, 148>104, 149>104, 149>105, 150>105	10
Fumarate	9.75	175>115, 176>116, 177>117, 178>118, 179>119	10
3PGA	9.78	185>79.05, 186>79.05, 187>79.05, 188>79.05	32
FBP	9.95	339>97.05, 340>97.05, 341>97.05, 342>97.05, 343>97.05, 344>97.05, 345>97.05	18
Citrate + Isocitrate*	10.04	191>111.1, 192>111.1, 192>112.1, 193>112.1, 193>113.1, 194>113.1, 194>114.1, 195>114.1, 195>115.1, 196>115.1, 196>116.1, 197>116.1	15
RuBP	10.09	309>97.05, 310>97.05, 311>97.05, 312>97.05, 313>97.05, 314>97.05	18
PEP	10.10	167>79.1, 168>79.1, 169>79.1, 170>79.1	13
PRPP	11.19	389>177.05, 390>177.05, 391>177.05, 392>177.05, 393>177.05, 394>177.05	21
1,3-BPG	11.20	265>167.05, 266>168.05, 267>169.05, 268>170.05	18
CoA	11.99	766>79, 767>79, 768>79, 769>79, 770>79, 771>79, 772>79, 773>79, 774>79, 775>79, 776>79, 777>79 778>79, 779>79, 780>79, 781>79, 782>79, 783>79, 784>79, 785>79, 786>79, 787>79	54
3HB-CoA	12.09	852.1>408.1, 853.1>408.1, 854.1>408.1, 855.1>408.1, 856.1>408.1	40
Acetyl-CoA	12.25	808.1>408.1, 809.1>408.1, 810.1>408.1	37
Succinyl-CoA	12.30	866.1>408.1, 867.1>408.1, 868.1>408.1, 869.1>408.1, 870.1>408.1	41
Crotonyl-CoA	13.24	834.1>408.1, 835.1>408.1, 836.1>408.1, 837.1>408.1, 838.1>408.1	36
Butyryl-CoA	13.34	836.1>408.1, 837.1>408.1, 838.1>408.1, 839.1>408.1, 840.1>408.1	37

[a]RT: retention time (min); [b]CE: collision energy (V).

*Could not be discriminated.
